# Coordination of XeF_2_ to Fluoridometal Cations: The Adduct Cations [PtF_3_(XeF_2_)_3_]^+^ and [PdF_3_(XeF_2_)_3_]^+^

**DOI:** 10.1021/acs.inorgchem.5c05940

**Published:** 2026-02-26

**Authors:** Klemen Motaln, Miha Virant, Matic Lozinšek

**Affiliations:** † Extreme Conditions Chemistry Laboratory (ECCL K2), 61790 Jožef Stefan Institute, Jamova cesta 39, 1000 Ljubljana, Slovenia; ‡ Jožef Stefan International Postgraduate School, Jamova cesta 39, 1000 Ljubljana, Slovenia

## Abstract

Crystals of the double salts [Xe_2_F_3_]­[MF_3_(XeF_2_)_3_]­[AsF_6_]_2_ (M = Pd, Pt), prepared from anhydrous HF solutions, were characterized by low-temperature single-crystal X-ray diffraction and Raman spectroscopy. The crystal structures reveal that the compounds contain the hitherto unobserved [MF_3_(XeF_2_)_3_]^+^ adduct cations, which differ from all previously identified examples of XeF_2_ coordination to a metal­(IV) center in their mononuclear and cationic nature, as well as in the coordination of multiple XeF_2_ ligands to a single metal­(IV) center. Quantum-chemical calculations were performed to gain insight into the bonding and electronic structure of the [MF_3_(XeF_2_)_3_]^+^ adduct cations, with the optimized gas-phase geometries showing good agreement with the experimental solid-state structures. The characterized [MF_3_(XeF_2_)_3_]^+^ adduct cations markedly extend the chemistry of XeF_2_–MF_4_ systems and represent rare, crystallographically characterized examples of XeF_2_ coordination to Pt^IV^ and Pd^IV^, a feature also believed to occur in the structure of the first discovered noble-gas compound, XePtF_6_. Moreover, the [MF_3_(XeF_2_)_3_]^+^ cations constitute the first examples of a new class of XeF_2_ coordination compounds, which are characterized by XeF_2_ ligation to a fluoridometal [MF*_*x*_*]^*n*+^ cation, thereby enabling further expansion of the coordination chemistry of noble-gas fluorides.

## Graphical abstract

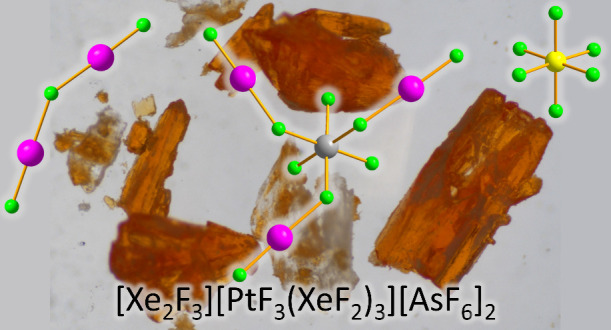

## Introduction

Xenon difluoride, XeF_2_, is arguably the most well-known noble-gas compound and, owing to its commercial availability, one of the most important synthetic precursors for xenon chemistry.^1^ The reactivity of XeF_2_ is typically governed by its pronounced Lewis basicity and fluoride-ion donor abilities.^2,3^ Upon interaction with Lewis acids or fluoride-ion acceptors, XeF_2_ forms a variety of complexes, adducts, and tight ion-pair salts (Figure 1),^4^ exhibiting varying degrees of ionization along the following pathway: XeF_2_ → XeF^+^ + F^–^.^5^ Compounds derived from metal-based Lewis acids can be qualitatively divided into three classes:

i) **[M** ^*n* **+**^ **(XeF**_**2**_ **)**_*m*_ **(AF**_*x*_^**–**^ **)**_*n*_ **] complexes**, formed by coordination of XeF_2_ to a “naked” metal cation M^*n*+^ derived from an M­(AF*_*x*_*)*_n_* salt with a weakly coordinating anion, for example homoleptic [Cu­(XeF_2_)_6_]­(RuF_6_)_2_, anion-bridged [Ni­(XeF_2_)_2_(RuF_6_)_2_],^6^ and anion- and XeF_2_-bridged [Ba­(XeF_2_)_4_(AsF_6_)_2_]·XeF_2_ (Figure 1a).^7^
ii) *m* **XeF**_**2**_ **·** *n* **MF**_*x*_ **and** *m* **XeF**_**2**_ **·** *n* **MO**_*x*_ **F**_*y*_ **Lewis acid–base adducts**, formed by interaction of XeF_2_ with moderately strong Lewis acids such as metal tetrafluorides and oxyfluorides, for example: XeF_2_·CrF_4_,^8^ 3XeF_2_·2MnF_4_, XeF_2_·MnF_4_,^9^ XeF_2_·CrOF_4_, XeF_2_·2CrOF_4_,^10^ XeF_2_·MoOF_4_, XeF_2_·WOF_4_, XeF_2_·2WOF_4_ (Figure 1b).^11^
iii) **[XeF]** ^**+**^ **tight ion-pair salts and [Xe**_**2**_ **F**_**3**_ **]** ^**+**^ **salts**, produced in reactions with strongly Lewis acidic metal or metalloid pentafluorides, for example: [XeF]­[AF_6_] (A = As, Nb, Ru, Sb, Ta, Pt, Bi), [XeF]­[A_2_F_11_] (A = Ru, Sb, Ta, Pt, Bi), and [Xe_2_F_3_]­[AF_6_] (A = As, Ru, Sb, Ta, Pt, Au, Bi) (Figure 1c).^1,4,12–15^

**Figure 1:**
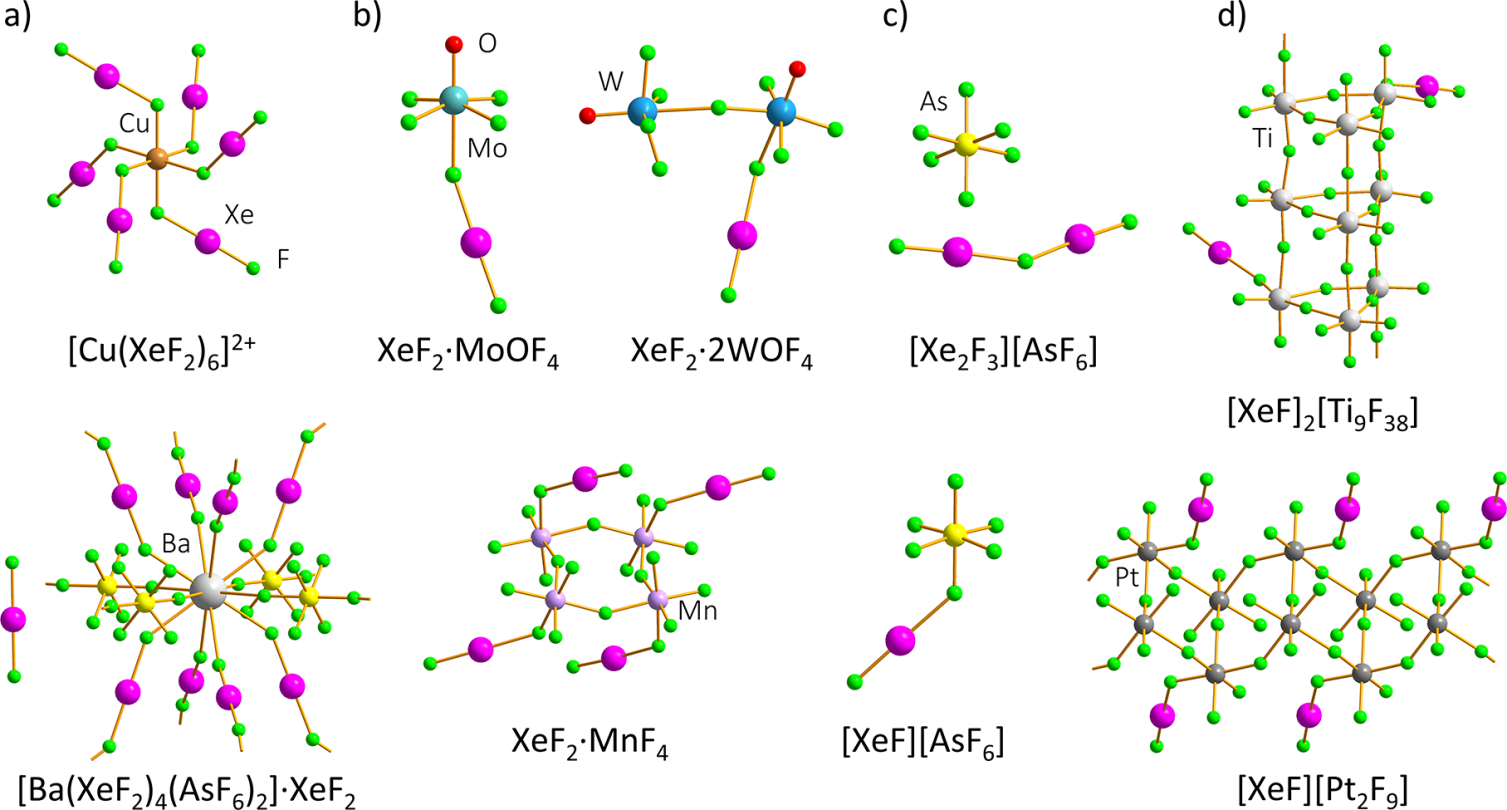
Structural diversity of compounds stemming from the interaction of XeF_2_ with metal-based (and metalloid-based) Lewis acids.^6,7,9,11,13,15–17^

However, there are borderline cases that may be classified as either class (ii) or class (iii). For example, 2XeF_2_·9TiF_4_ and XeF_2_·2MF_4_ (M = Mn, Pd, Pt) exhibit sufficient ionization of XeF_2_ coordinated to the metal­(IV) center to justify ionic formulations of [XeF]^+^_2_[Ti_9_F_38_]^2–^ and [XeF]^+^[M_2_F_9_]^–^ (M = Mn, Pd, Pt), respectively (Figure 1d).^9,16,17^ In contrast, crossover species between classes (i) and (ii), such as [M^*n*+^F*_p_* (XeF_2_)*_m_*]^(*n*−*p*)+^(AF*_*x*_* ^–^)_*n*−*p*_, in which the metal center is coordinated by both XeF_2_ and F^–^ ligands, have not been reported. Comparable linkages have been observed in the coordination chemistry of the related KrF_2_, as the crystal structures of [μ-FHg­(μ_3_-FKrF)_1.5_(FKrF)_0.5_AsF_6_]_2_ and [μ_3_-FHg­(μ_3_-FKrF)_0.5_(FKrF)_1.5_AsF_6_]_2_ were shown to consist of extended three-dimensional networks incorporating bridging F^–^ and KrF_2_ units which ligate Hg^2+^ centers.^18^

In this work, the first examples of discrete [M^*n*+^F*_p_* (XeF_2_)*_m_*]^(*n*−*p*)+^ species are reported, represented by the unique [MF_3_(XeF_2_)_3_]^+^ (M = Pt, Pd) adduct cations, which were isolated as brightly colored crystals of the corresponding [Xe_2_F_3_]­[MF_3_(XeF_2_)_3_]­[AsF_6_]_2_ double salts. Despite the fact that XeF_2_ coordination to platinum constitutes a defining structural feature of the first discovered noble-gas compound, XePtF_6_ (XeF_2_·PtF_4_),^19,20^ the structural chemistry of the XeF_2_–PtF_4_ system and the related^21^ XeF_2_–PdF_4_ system remains poorly explored. The *fac*-[M^IV^F_3_(XeF_2_)_3_]^+^ adduct cations described herein represent only the second instance of crystallographically characterized species featuring XeF_2_ coordination to platinum and palladium, following the recently structurally characterized XeF_2_·2PtF_4_ and XeF_2_·2PdF_4_ adducts, whose structures were elucidated by 3D electron diffraction.^17^ The newly discovered [MF_3_(XeF_2_)_3_]^+^ (M = Pt, Pd) adduct cations thus offer additional insights into these historically important systems.

## Results and Discussion

### Synthesis

The title compounds were synthesized in anhydrous HF (aHF) by reactions of the appropriate binary fluorides in the molar ratios observed in the final products (XeF_2_/MF_4_/AsF_5_ = 5:1:2) (eqs 1, 2).

$$5{\mathrm{XeF}}_{2}+{\mathrm{PtF}}_{4}+2{\mathrm{AsF}}_{5}\overset{\mathrm{aHF}}{\to }x[{\mathrm{Xe}}_{2}F_{3}][{\mathrm{PtF}}_{3}({\mathrm{XeF}}_{2}{)}_{3}]{\left[{\mathrm{AsF}}_{6}\right]}_{2}(oP256)+y[{\mathrm{Xe}}_{2}F_{3}]\left[{\mathrm{AsF}}_{6}\right]$$ 1

$$5{\mathrm{XeF}}_{2}+{\mathrm{PdF}}_{4}+2{\mathrm{AsF}}_{5}\overset{\mathrm{aHF}}{\to }x\left[{\mathrm{Xe}}_{2}F_{3}\right]\left[{\mathrm{PdF}}_{3}{\left({\mathrm{XeF}}_{2}\right)}_{3}\right]{\left[{\mathrm{AsF}}_{6}\right]}_{2}+y\left[{\mathrm{Xe}}_{2}F_{3}\right]\left[{\mathrm{AsF}}_{6}\right]+z\left({\mathrm{XeF}}_{2}\cdot 2{\mathrm{PdF}}_{4}\right)$$ 2

To the best of our knowledge, this approach represents a rare targeted investigation of XeF_2_ reactivity in a system containing both Lewis-acidic tetra- and pentafluorides. The fluorobasic conditions established by the excess of XeF_2_ ensured partial dissolution of the otherwise aHF-insoluble metal tetrafluorides, resulting in yellow-colored solutions, into which AsF_5_ was subsequently dosed. Slow crystallization induced by solvent evaporation afforded amber-colored crystals (Figure S1), which were isolated and characterized by low-temperature (LT) single-crystal X-ray diffraction (SCXRD) and LT Raman spectroscopy. The formation of [Xe_2_F_3_]­[MF_3_(XeF_2_)_3_]­[AsF_6_]_2_ single crystals was invariably accompanied by the formation of colorless crystals of [Xe_2_F_3_]­[AsF_6_], which were identified by Raman spectroscopy and SCXRD. Additional reaction pathways were explored by reacting a 2-fold molar excess of [XeF]­[AsF_6_] with K_2_PtF_6_, or by combining [XeF]­[AsF_6_], XeF_2_·PdF_4_, and XeF_2_ while maintaining a XeF_2_/MF_4_/AsF_5_ ratio of 5:1:2 (see the Experimental Section). In both cases, the title compounds were again obtained; however, in the former reaction, a triclinic polymorph of [Xe_2_F_3_]­[PtF_3_(XeF_2_)_3_]­[AsF_6_]_2_ was isolated (eq 3).

$$2\left[\mathrm{XeF}\right]\left[{\mathrm{AsF}}_{6}\right]+K_{2}{\mathrm{PtF}}_{6}\overset{\mathrm{aHF}}{\to }x\left[{\mathrm{Xe}}_{2}F_{3}\right]\left[{\mathrm{PtF}}_{3}{\left({\mathrm{XeF}}_{2}\right)}_{3}\right]{\left[{\mathrm{AsF}}_{6}\right]}_{2}\left(aP256\right)+y{\mathrm{KAsF}}_{6}+z\left(n{\mathrm{XeF}}_{2}\cdot {\mathrm{PtF}}_{4}\right)$$ 3

### Crystal Structures

Structural analysis revealed that [Xe_2_F_3_]­[PtF_3_(XeF_2_)_3_]­[AsF_6_]_2_, synthesized from the binary fluorides XeF_2_, PtF_4_, and AsF_5_, crystallizes in the orthorhombic *P*2_1_2_1_2_1_ space group, whereas the polymorph prepared by the reaction of [XeF]­[AsF_6_] with K_2_PtF_6_ crystallizes in the lower-symmetry triclinic $P\overset{-}{1}$ space group. The two polymorphic forms are henceforth referred to by their Pearson symbols as *oP*256 and *aP*256, respectively. In contrast, crystal structure determination of [Xe_2_F_3_]­[PdF_3_(XeF_2_)_3_]­[AsF_6_]_2_ revealed that it is not isotypic with either of the [Xe_2_F_3_]­[PtF_3_(XeF_2_)_3_]­[AsF_6_]_2_ polymorphs and instead crystallizes in the monoclinic *P*2_1_ space group (Tables 1 and S1). Tables 1 and 2 contain the summary of crystal data and refinement results, and the selected bond lengths and angles observed in the crystal structures, respectively, whereas complete crystallographic tables are provided in the Supporting Information (Tables S1–S4).

**Table 1:** Summary of Crystal Data and Refinement Results for [Xe_2_F_3_]­[PtF_3_(XeF_2_)_3_]­[AsF_6_]_2_(*oP*256) [Xe_2_F_3_]­[PtF_3_(XeF_2_)_3_]­[AsF_6_]_2_(*aP*256), and [Xe_2_F_3_]­[PdF_3_(XeF_2_)_3_]­[AsF_6_]_2_

	[Xe_2_F_3_][PtF_3_(XeF_2_)_3_][AsF_6_]_2_(*oP*256)	[Xe_2_F_3_][PtF_3_(XeF_2_)_3_][AsF_6_]_2_(*aP*256)	[Xe_2_F_3_][PdF_3_(XeF_2_)_3_][AsF_6_]_2_
space group	*P*2_1_2_1_2_1_	$P\overset{-}{1}$	*P*2_1_
*a* (Å)	11.86164(10)	12.92922(7)	6.53000(4)
*b* (Å)	16.48399(13)	13.07591(6)	11.83284(6)
*c* (Å)	23.8108(2)	27.91602(13)	14.93053(9)
α (°)	90	83.6237(4)	90
β (°)	90	79.6369(4)	90.0233(5)
γ (°)	90	75.7149(5)	90
*V* (Å^3^)	4655.66(7)	4487.97(4)	1153.658(11)
*Z*	8	8	2
*M*_w_ (g mol^–1^)	1457.43	1457.43	1368.74
*D*_calcd_ (g cm^–3^)	4.159	4.314	3.940
μ (mm^–1^)	8.639	8.962	5.838
*R*_1_	0.0389	0.0319	0.0320
*wR*_2_	0.1001	0.0811	0.0797
Δρ_min__,_ _max_ (e Å^–3^)	–1.958, 5.733	–2.818, 4.386	–1.959, 1.990

**Table 2:** Selected Bond Lengths (Å) and Angles (°) for the Crystal Structures of [Xe_2_F_3_]­[PtF_3_(XeF_2_)_3_]­[AsF_6_]_2_ and [Xe_2_F_3_]­[PdF_3_(XeF_2_)_3_]­[AsF_6_]_2_

	**[Xe**_**2**_ **F**_**3**_ **][PtF**_**3**_ **(XeF**_**2**_ **)**_**3**_ **][AsF**_**6**_ **]**_**2**_ **(** *oP* **256)**	**[Xe**_**2**_ **F**_**3**_ **][PtF**_**3**_ **(XeF**_**2**_ **)**_**3**_ **][AsF**_**6**_ **]**_**2**_ **(** *aP* **256)**	**[Xe**_**2**_ **F**_**3**_ **][PdF**_**3**_ **(XeF**_**2**_ **)**_**3**_ **][AsF**_**6**_ **]**_**2**_
Xe–F_t_	1.888(6)–1.921(6)	1.898(3)–1.918(3)	1.918(4)–1.923(4)
Xe–F_b_(M)	2.182(5)–2.211(5)	2.179(3)–2.214(2)	2.143(4)–2.178(4)
Xe–F_t_ ^a^	1.906(5)–1.929(5)	1.908(3)–1.923(3)	1.915(4), 1.916(4)
Xe–F_b_(Xe)	2.125(5)–2.174(5)	2.157(3)–2.188(3)	2.142(4), 2.162(4)
M–F_t_	1.881(5)–1.898(5)	1.882(3)–1.897(2)	1.842(5)–1.860(4)
M–F_b_(Xe)	1.978(5)–1.996(5)	1.981(3)–2.002(2)	1.975(4)–1.990(4)
As–F	1.657(13)–1.786(13)	1.704(3)–1.737(3)	1.714(4)–1.740(4)
F–Xe–F	175.9(2)–176.8(2)	173.35(14)–179.40(12)	177.2(2)–178.2(2)
F–Xe–F^a^	177.1(3)–178.9(3)	178.11(15)–179.36(14)	178.1(2), 179.36(19)
Xe–F_b_–Xe^a^	139.9(3), 140.3(3)	127.10(13)–134.65(15)	133.11(19)
*cis*-F–M–F	87.0(2)–93.3(2)	85.56(12)–92.53(12)	87.9(2)–92.6(3)
*trans-*F–M–F	178.3(2)–179.6(2)	177.12(12)–179.00(12)	178.16(19)–179.5(2)
*cis*-F–As–F	79.7(8)–98.3(8)	88.11(17)–91.72(15)	87.5(2)–91.2(3)
*trans-*F–As–F	169.0(11)–179.3(4)	177.69(17)–179.73(17)	177.4(2)–179.7(3)

a Pertaining to the bonds in the [Xe_2_F_3_]^+^ cations.

In all structures, the asymmetric unit is comprised of isolated [Xe_2_F_3_]^+^, [MF_3_(XeF_2_)_3_]^+^, and [AsF_6_]^−^ ions, with all atoms occupying general positions (Figures 2,3 and S2–S6). The M^IV^F_3_^+^ fragment is coordinated by three XeF_2_ ligands in a *fac* arrangement, resulting in a slightly distorted octahedral coordination environment around the M^IV^ center. These compounds, which feature three XeF_2_ ligands coordinated to a single metal­(IV) center, currently represent the highest number of XeF_2_ ligands observed for any metal­(IV) species. They also constitute a unique case in which XeF_2_ coordination to a metal­(IV) center results in a mononuclear complex. In contrast, the other known compounds exhibiting XeF_2_ coordination to metal­(IV) centers predominantly adopt polymeric structures composed of interconnected [MF_6_] octahedra, forming chains (XeF_2_·CrF_4_ and 3XeF_2_·2MnF_4_),^8,9^ double chains (XeF_2_·2MnF_4_, XeF_2_·2PdF_4_, and XeF_2_·2PtF_4_)^9,17^ (Figure 1d), columnar motifs ([XeF]_2_[Ti_9_F_38_])^16^ (Figure 1d), or layers (XeF_2_·2CrF_4_).^22^ The sole exception within this family of compounds is XeF_2_·MnF_4_, whose structure comprises discrete tetrameric ring units (Figure 1b). Across all of these structures, each metal­(IV) center is coordinated by no more than one XeF_2_ ligand.

**Figure 2:**
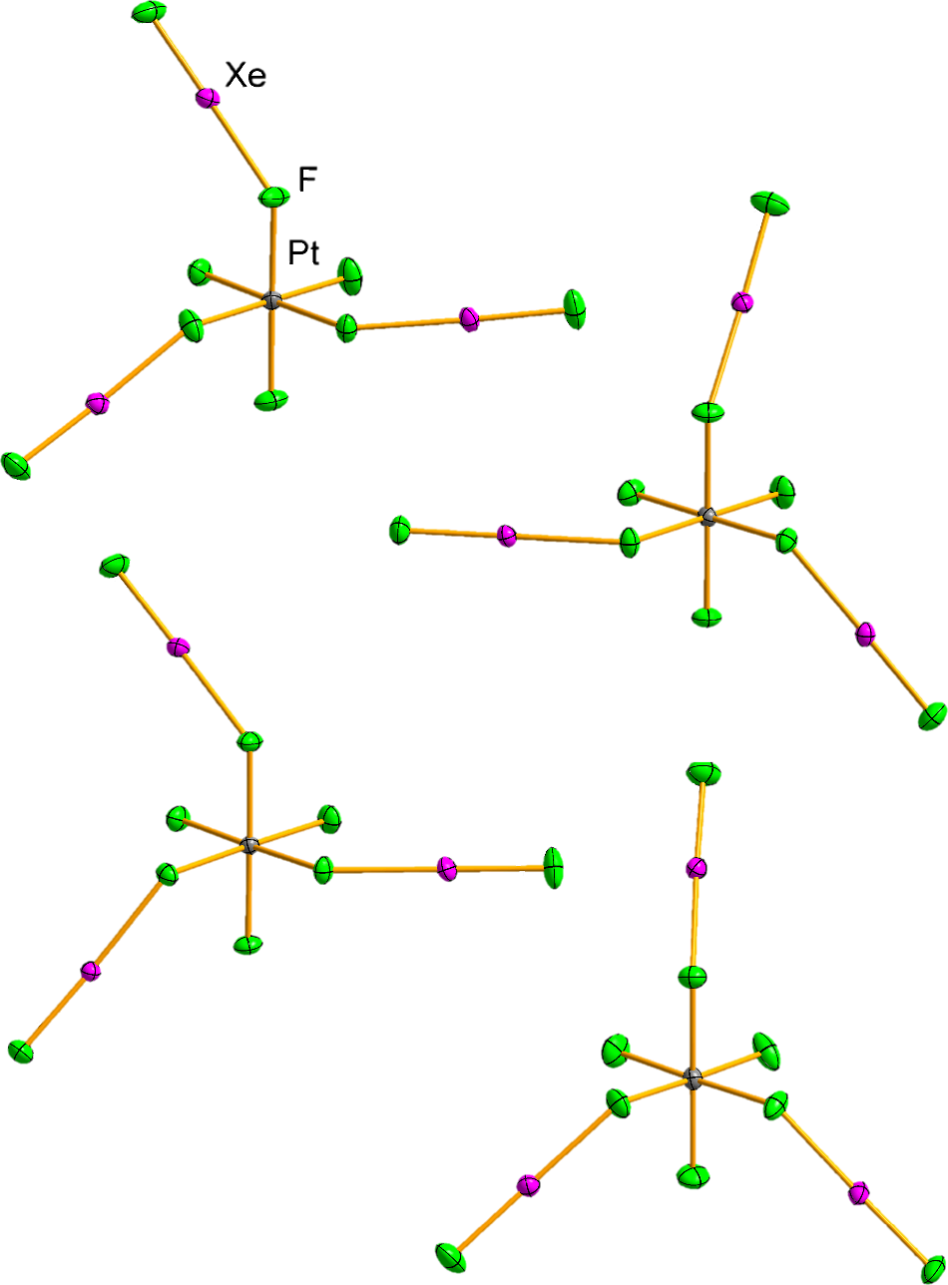
Four crystallographically independent [PtF_3_(XeF_2_)_3_]^+^ cations in the crystal structure of [Xe_2_F_3_]­[PtF_3_(XeF_2_)_3_]­[AsF_6_]_2_(*aP*256). Displacement ellipsoids are drawn at the 50% probability level.

**Figure 3:**
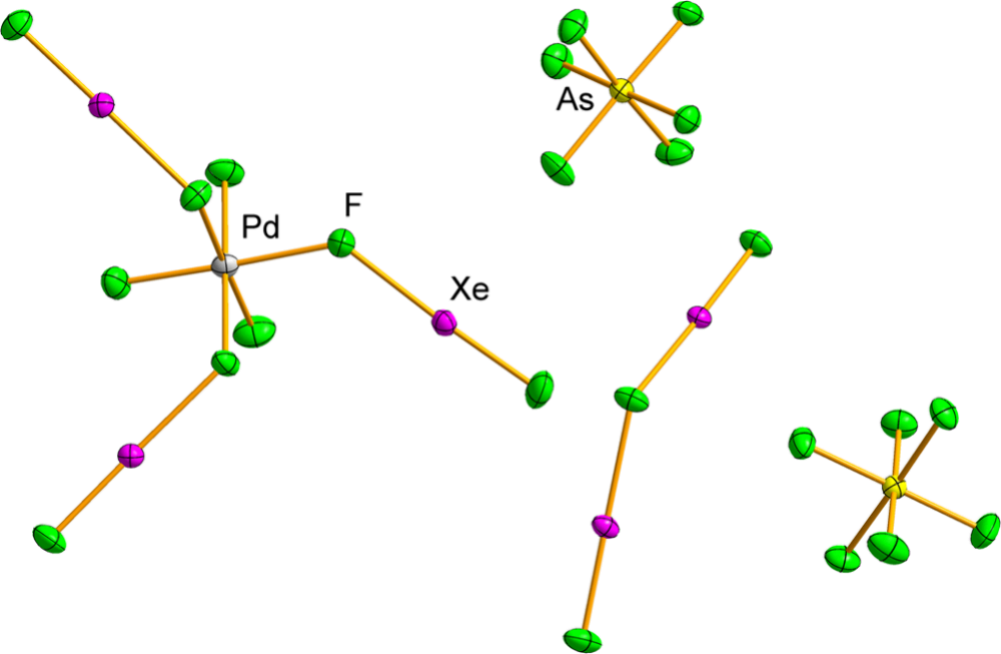
Asymmetric unit of the [Xe_2_F_3_]­[PdF_3_(XeF_2_)_3_]­[AsF_6_]_2_ crystal structure. Displacement ellipsoids are drawn at the 50% probability level.

The geometry of the coordinated XeF_2_ ligands in the [PtF_3_(XeF_2_)_3_]^+^ and [PdF_3_(XeF_2_)_3_]^+^ adduct cations is perturbed, showing slight deviations of the F–Xe–F angles from linearity [175.9(2)–176.8(2)° in *oP*256, 173.35(14)–179.35(12)° in *aP*256, and 177.2(2)–178.2(2)° in the Pd compound] and a pronounced elongation of the bridging Xe–F_b_ bonds, accompanied by shortening of the terminal Xe–F_t_ bonds. The Xe–F_t_ and Xe–F_b_ bond lengths in the [PdF_3_(XeF_2_)_3_]^+^ cation span the ranges of 1.918(4)–1.923(4) Å and 2.143(4)–2.178(4) Å, respectively, with average values of 1.920 and 2.159 Å. These distances indicate that the XeF_2_ moiety displays an intermediate degree of ionization, comparable to that observed in some XeF_2_–MF_4_ adducts, namely 3XeF_2_·2MnF_4_, XeF_2_·MnF_4_,^9^ and XeF_2_·CrF_4_.^8^ For comparison, the Xe–F bond length of the centrosymmetric XeF_2_ molecule (*D*_∞h_) in the solid state is 1.999(4) Å.^13^ In the case of the [PtF_3_(XeF_2_)_3_]^+^ cations, a greater variability in the Xe–F bond lengths is observed. The Xe–F_t_ bonds measure 1.888(6)–1.921(6) Å and 1.898(3)–1.918(3) Å, whereas the Xe–F_b_ bonds measure 2.182(5)–2.211(5) Å and 2.179(3)–2.214(2) Å in the *oP*256 and *aP*256 polymorphs, respectively. Examination of the average values of the Xe–F_t_ and Xe–F_b_ bond lengths, which amount to 1.908 Å and 2.196 Å in the *oP*256 polymorph, and 1.908 Å and 2.200 Å in the *aP*256 polymorph, reveals a pronounced distortion of the geometry of the coordinated XeF_2_ ligands. The mean values are comparable to the bond lengths observed in the [XeF]­[BiF_6_] tight ion-pair salt [Xe–F_t_: 1.913(7) Å; Xe–F_b_: 2.204(7) Å],^13^ providing evidence for the substantial fluoride-ion affinity of the [PtF_3_]^+^ cation. Nevertheless, the fluoride-ion affinity of these cations is less pronounced than that exhibited by the polymeric fluoridoplatinate­(IV) and fluoridopalladate­(IV) units present in the crystal structures of XeF_2_·2MF_4_ (M = Pt, Pd), where Xe–F_b_ bond lengths exceeding 2.3 Å are observed.^17^ The Pt–F_t_ [*oP*256: 1.881(5)–1.898(5) Å; *aP*256: 1.882(3)–1.897(2) Å] and Pd–F_t_ bond lengths [1.842(5)–1.860(5) Å] show good agreement with the values observed in the crystal structures of XeF_2_·2PtF_4_ [1.863(7)–1.905(11) Å] and XeF_2_·2PdF_4_ [1.844(4)–1.866(4) Å], respectively. Conversely, the bridging Pt–F_b_(Xe) bond distances [*oP*256: 1.978(5)–1.996(5) Å; *aP*256: 1.981(3)–2.002(2) Å] and Pd–F_b_(Xe) [1.975(4)–1.990(4) Å] are notably elongated in comparison to their counterparts observed in the crystal structures of XeF_2_·2PtF_4_ [1.932(8) Å] and XeF_2_·2PdF_4_ [1.894(4) Å], respectively.^17^ In all three crystal structures, a number of inter- and intramolecular Xe···F contacts are formed, with distances shorter than the corresponding sum of the van der Waals radii 3.74 Å (Tables S5–S7).^23,24^ These contacts contribute to stabilization of the crystal packing and influence the marked conformational differences observed among the crystallographically independent [PtF_3_(XeF_2_)_3_]^+^ and [PdF_3_(XeF_2_)_3_]^+^ adduct cations (Figure S7). In the crystal structures, the adduct cations possess *C*_1_ point-group symmetry and are therefore chiral. As [Xe_2_F_3_]­[PtF_3_(XeF_2_)_3_]­[AsF_6_]_2_(*oP*256) and [Xe_2_F_3_]­[PdF_3_(XeF_2_)_3_]­[AsF_6_]_2_ crystallize in Sohncke space groups, only one enantiomeric form of each cation is present in each crystal structure. Conversely, both enantiomers of the cation are present in the centrosymmetric $P\overset{-}{1}$ crystal structure of [Xe_2_F_3_]­[PtF_3_(XeF_2_)_3_]­[AsF_6_]_2_(*aP*256).

The second cationic species present in these double salts are the planar, V-shaped [Xe_2_F_3_]^+^ cations. Interestingly, the Xe–F_b_–Xe angles determined in the crystal structures of [Xe_2_F_3_]­[PtF_3_(XeF_2_)_3_]­[AsF_6_]_2_(*aP*256) [127.10(13)–134.65(15)°] and [Xe_2_F_3_]­[PdF_3_(XeF_2_)_3_]­[AsF_6_]_2_ [133.11(19)°] (Figures S3 and 3) are the least obtuse angles observed for [Xe_2_F_3_]^+^ cations to date. Previously reported fluoride-bridge angles span the range from 139.8(8)° in trigonal [Xe_2_F_3_]­[AsF_6_][Bibr ref15] to 164.3(3)° observed in the crystal structure of [Xe_2_F_3_]­[Ti_8_F_33_].^16^ On the other hand, the Xe–F_b_–Xe angles observed in the crystal structure of [Xe_2_F_3_]­[PtF_3_(XeF_2_)_3_]­[AsF_6_]_2_(*oP*256) are noticeably more open [139.9(3)° and 140.3(3)°]. As quantum-chemical calculations have previously shown that the vibrational mode corresponding to the bending deformation of the Xe–F_b_–Xe angle is very low in frequency, the large variability of these angles has been ascribed to crystal-packing effects.^15^

### Computational Results

To investigate the electronic structures of the isolated [MF_3_(XeF_2_)_3_]^+^ cations, their geometries were optimized using the DFT method PBE0-D3/aug-cc-pVQZ­(-PP) as implemented in the *ORCA* software (version 6.0.0).^25^ The resulting stationary points were confirmed to be true minima on the potential energy surface with no imaginary frequencies (Table S8). In the absence of additional interactions, the isolated cations examined in the computational analysis converged to a slightly distorted *C*_3v_ symmetry (Figure 4, Tables S9 and S10). A comparison of the experimentally determined and calculated bond lengths demonstrates good agreement, with deviations not exceeding 0.6% (Table S11 and S12). However, greater discrepancies between the experimental and calculated structures are observed in the conformation of the cations. In particular, the Xe–F_b_–M angles are, on average, more acute in the calculated geometries than in the experimental structures (Tables S11 and S12). Moreover, although the Xe–F_b_–M–F_t_’ (where F_t_′ denotes the terminal F atom nearest to Xe) torsion angles observed in the crystal structures span a wide range of values (Tables S2–S4), the calculated geometries converge to torsion angles that correspond to staggered conformations (Tables S11 and S12).^13^

**Figure 4:**
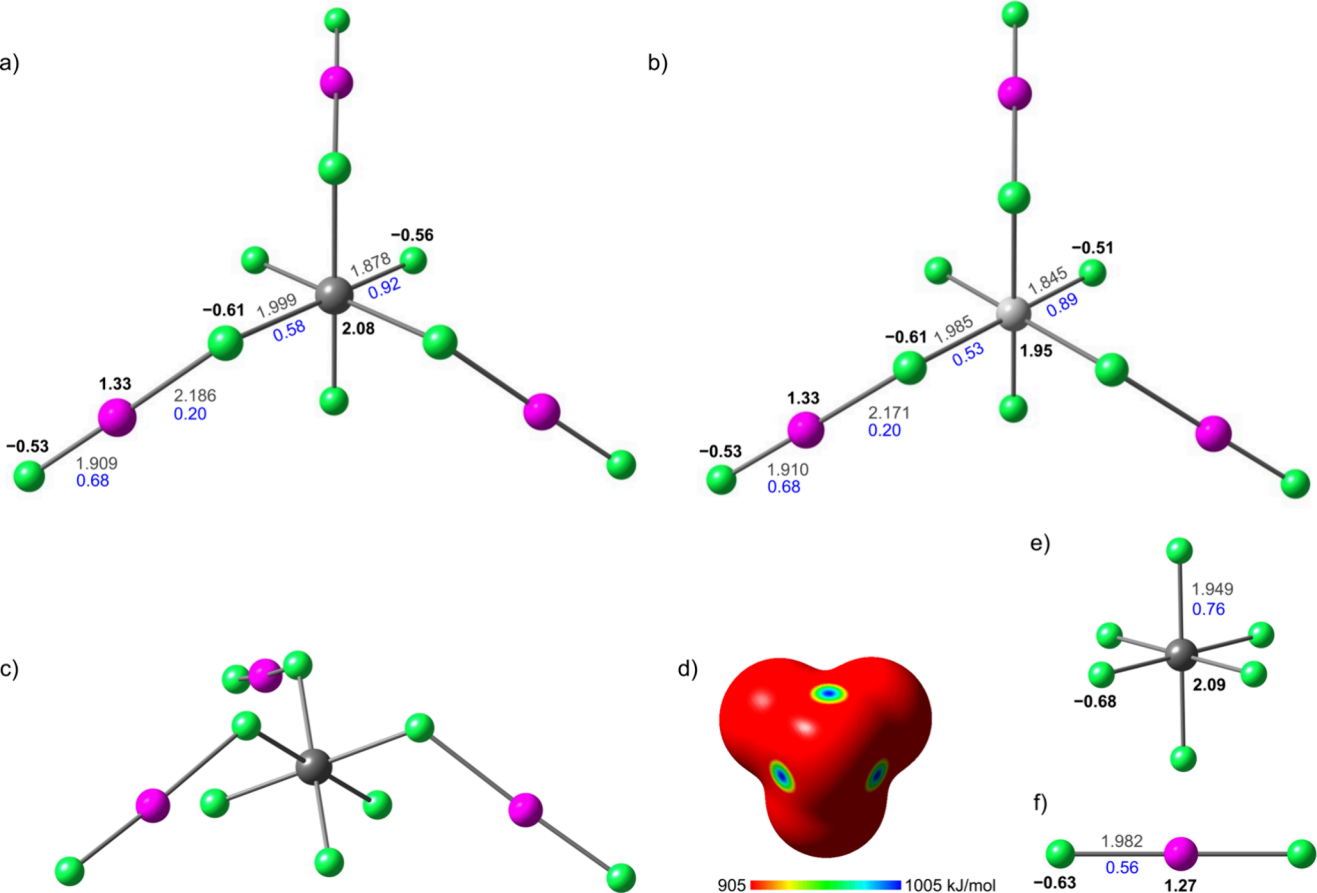
Calculated gas-phase geometries of (a) [PtF_3_(XeF_2_)_3_]^+^ and (b) [PdF_3_(XeF_2_)_3_]^+^ cations; (c) an alternative view of [PtF_3_(XeF_2_)_3_]^+^ cation; (d) molecular electrostatic potential surface (MEPS) of [PtF_3_]^+^ fragment (top 10% of the positive electrostatic potential range); and optimized gas-phase geometries of (e) [PtF_6_]^2–^ anion and (f) free XeF_2_, included for comparison. Bond distances (Å) are indicated in gray, AIM charges are listed in black, and Mayer bond orders are given in blue.

The studied [MF_3_(XeF_2_)_3_]^+^ adduct cations can be described in terms of two limiting structures: one featuring neutral XeF_2_ ligands coordinated to a [MF_3_]^+^ cation, and the other corresponding to complete ionization into [XeF]^+^ cations coordinated to a [MF_6_]^2–^ anion.

Unbound, centrosymmetric XeF_2_ is characterized by negative atoms-in-molecules (AIM) charges on the fluorine atoms (−0.63), a positive charge on the xenon atom (+1.27), and equal Mayer bond orders (0.56) for both Xe–F bonds (1.982 Å; Figure 4, Table S13). Upon coordination, XeF_2_ becomes polarized, developing a net positive charge (+0.20). The terminal fluorine atom (−0.53) becomes less negative and the xenon atom (+1.33) becomes more positive, accompanied by shortening of the terminal Xe–F_t_ bond (1.909 Å) and an increase in the corresponding Mayer bond order (0.68). In contrast, the charge on the bridging fluorine atoms remains nearly unchanged (−0.61), while the bridging Xe–F_b_ bonds are significantly elongated (Pt: 2.186 Å; Pd: 2.171 Å), resulting in a decrease in the corresponding Mayer bond orders (0.20). These changes are attributed to the transfer of electron density from XeF_2_ toward regions of positive electrostatic potential on the [MF_3_]^+^ fragments, which display pronounced σ-holes along the extensions of the M–F bonds (Figures 4, S8, S9 and Table S14). The coordination of XeF_2_ ligands to the MF_3_^+^ cation can therefore be described as a regium-bonding interaction.^26^

The AIM charges of the metal centers in the adduct cations (Pt: +2.08; Pd: +1.95) are nearly identical to those calculated for the isolated [PtF_6_]^2–^ (+2.09) and [PdF_6_]^2–^ (+1.97) anions, whereas the fluorine atoms of the anions carry more negative charge (Pt: −0.68; Pd: −0.66) than either the bridging (−0.61) or terminal F atoms (Pt: −0.56; Pd: −0.51) in the adduct cations (Figure 4, Tables S15, S16). The total charge of the [MF_6_] fragments (−1.41) is less negative than that of the isolated [MF_6_]^2–^ anions (−2.00), consistent with the positive charge developed on the coordinated XeF_2_ ligands (+0.59). The Mayer bond orders of the M–F_t_ bonds in the adduct cations (Pt: 0.92; Pd: 0.89) are significantly higher than those calculated for the M–F bonds in the [MF_6_]^2–^ anions (Pt: 0.76; Pd: 0.70), whereas the M–F_b_ bond orders in the adduct cations are smaller (Pt: 0.58; Pd: 0.53).

The topological parameters obtained from the quantum theory of atoms in molecules (QTAIM) analysis^27^ (Table S17) further corroborate the polarization of XeF_2_. Specifically, the Xe–F_b_ bond exhibits a reduced covalent character relative to uncoordinated XeF_2_, as indicated by a lower electron density at the bond critical point (ρ­(*r*)), a smaller Laplacian of electron density (∇^2^ρ­(*r*)), and a less negative local energy density (*H*(*r*)). In contrast, the Xe–F_t_ bond shows an enhanced covalent character, reflected in higher ρ­(*r*) values, a larger ∇^2^ρ­(*r*), and a more negative *H*(*r*). The M–F_b_ interactions, characterized by low ρ­(*r*) values, small positive ∇^2^ρ­(*r*), and slightly negative *H*(*r*) values, are consistent with predominantly electrostatic σ-hole–type interactions.^11^

### Vibrational Spectroscopy

Low-temperature Raman spectra of the compounds, recorded at 100 K, exhibit the strongest bands in the Xe–F_t_ stretching region (580–620 cm^–1^; Figure 5, Table S18). Given that stretching modes of both [Xe_2_F_3_]^+^ and coordinated, partially ionized XeF_2_ species are expected to occur in this region,^9,15,28,29^ assignment of the observed bands to the respective vibrational modes is challenging. The spectra of [Xe_2_F_3_]­[MF_3_(XeF_2_)_3_]­[AsF_6_]_2_ exhibit similarities to the Raman spectrum of [Xe_2_F_3_]­[AsF_6_], as evidenced by the nearly unchanged positions of the ν_1_ (681, 682 cm^–1^) and ν_5_ bands (369, 370 cm^–1^) of the [AsF_6_]^−^ anion in comparison to the Raman spectrum of [Xe_2_F_3_]­[AsF_6_], in which these bands are located at 681 and 367 cm^–1^, respectively (Figures S10 and S11).^28^ Another prominent feature of the spectra is the presence of medium-strong bands in the 630–650 cm^–1^ region. These bands correspond reasonably well to the calculated in-phase and out-of-phase M–F_t_ stretching modes of the [MF_3_(XeF_2_)_3_]^+^ cations (Table S8, Figure S10, S11). The ATR-IR spectra of samples containing [Xe_2_F_3_]­[MF_3_(XeF_2_)_3_]­[AsF_6_]_2_ double salts and the spectra of pure [Xe_2_F_3_]­[AsF_6_] and KAsF_6_, are provided in the Supporting Information (Figures S12 and S13).

**Figure 5:**
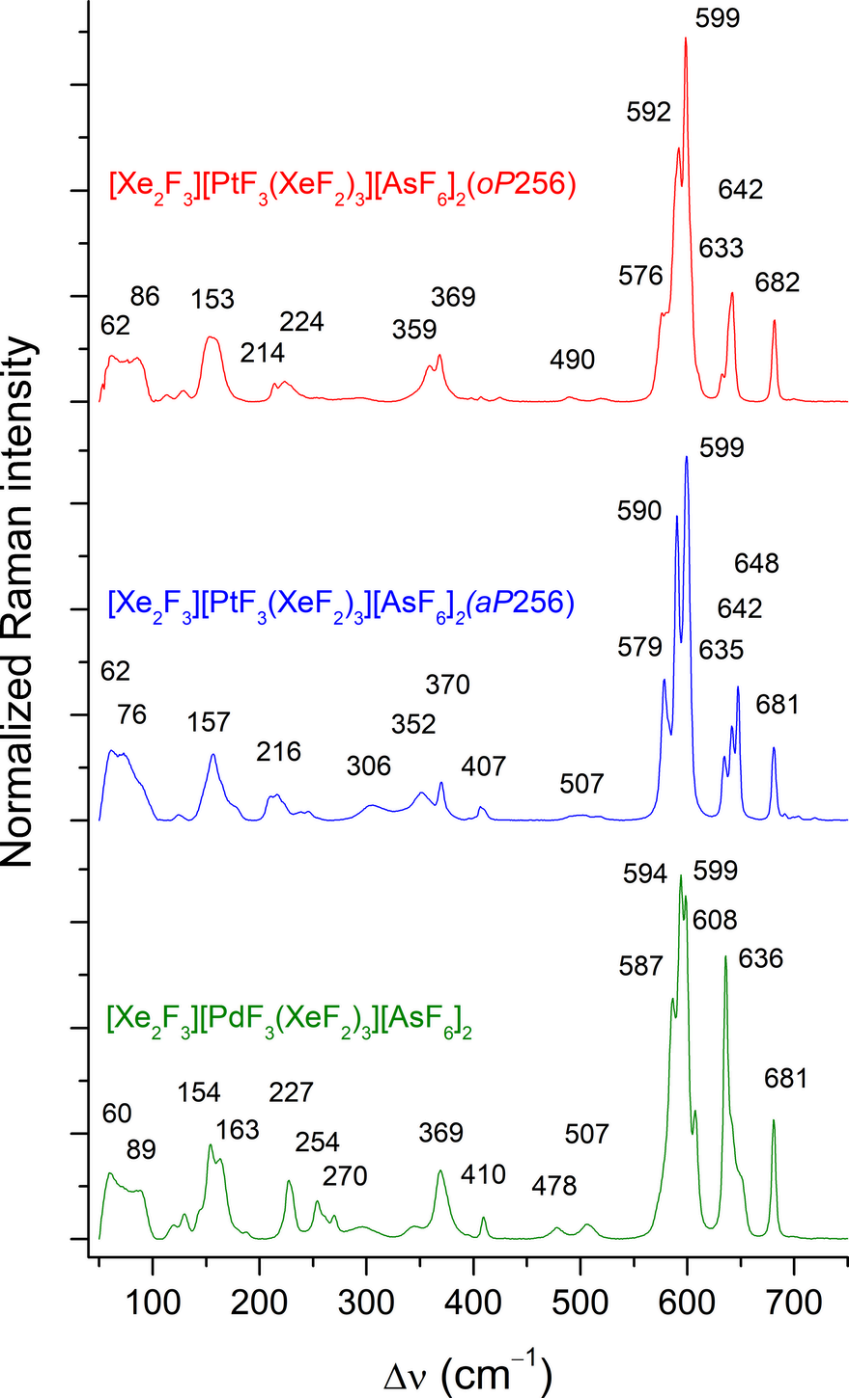
Raman spectra of the two polymorphs of [Xe_2_F_3_]­[PtF_3_(XeF_2_)_3_]­[AsF_6_]_2_ and [Xe_2_F_3_]­[PdF_3_(XeF_2_)_3_]­[AsF_6_]_2_ recorded at 100 K using 785 nm excitation.

## Conclusions

In summary, two polymorphic forms of [Xe_2_F_3_]­[PtF_3_(XeF_2_)_3_]­[AsF_6_]_2_, as well as [Xe_2_F_3_]­[PdF_3_(XeF_2_)_3_]­[AsF_6_]_2_, were synthesized and characterized by SCXRD, vibrational spectroscopy, and quantum-chemical calculations. The novel [MF_3_(XeF_2_)_3_]^+^ adduct cations present in the crystal structures of these double salts provide rare, structurally characterized examples of XeF_2_ coordination to platinum­(IV) and palladium­(IV) centers and represent the highest number of XeF_2_ ligands bound to a single metal­(IV) center observed to date. These findings highlight the substantial structural diversity exhibited by species in which XeF_2_ ligates a tetravalent metal center.

The adduct cations identified in this study define a new class of coordination complexes in which XeF_2_ serves as a ligand to a fluoridometal cation, thereby broadening the scope of XeF_2_ coordination chemistry. Moreover, the results indicate that probing the reactivity of XeF_2_ in systems containing multiple high-valent, Lewis-acidic fluorides can lead to the formation of novel species. This observation underscores the potential for systematic exploration across the broad landscape of mixed MF_4_/AF_5_–XeF_2_ systems, a largely unexplored domain with a strong potential for advancing this emerging class of compounds.

## Experimental Section

CAUTION: *Anhydrous HF, F*_*2*_ *, and AsF*_*5*_ *are highly toxic volatile substances and must be handled with great care in a well-ventilated fume hood while wearing appropriate personal protective equipment at all times. XeF*_*2*_ *and its derivatives are potent oxidizing fluorinating agents and release HF upon hydrolysis. Work with such chemicals should be undertaken only by properly trained experimentalists and the access to proper treatment procedures in the event of exposure must be ensured.* ^30–32^

### General

All experimental work on the compounds described herein was conducted under strictly anhydrous conditions. Manipulation of volatile materials was performed on a custom-built, fluorine-resistant vacuum line constructed from nickel, copper, poly­(tetrafluoroethylene) (PTFE), and fluorinated ethylene propylene (FEP). Solid materials were handled in a glovebox (Vigor SG1200/750E–SG1500/750E) under an inert nitrogen atmosphere, with moisture levels maintained below 1 ppm at all times.

The syntheses of [Xe_2_F_3_]­[MF_3_(XeF_2_)_3_]­[AsF_6_]_2_ compounds were carried out in h-shaped reaction vessels, constructed from two FEP tubes (6 mm i.d. × 8 mm o.d.) and equipped with either a brass-encased PTFE valve or an aluminum-encased poly­(chlorotrifluoroethylene) (PCTFE) valve. The FEP tubes were heat-sealed at one end and heat-flared at the other, with one tube bent at 90° angle. The two tubes were connected to a PTFE T-section via two male-to-male PTFE connectors, with the angled side arm positioned perpendicular to the main arm. Prior to use, the reaction vessels and PTFE-coated magnetic stirring bars were passivated with 300–500 Torr of F_2_ for a minimum of 8 h.

### Starting Materials

Fluorine gas (Solvay Fluor, 98–99%) was used as supplied. Residual moisture was removed from commercial anhydrous HF (Linde, 99.995%) by condensing it into an FEP vessel containing K_2_NiF_6_ (Advance Research Chemicals, 99.9%). XeF_2_ was synthesized by UV irradiation of a xenon (Messer, 99.99%) and fluorine mixture in a thoroughly dried glass bulb, employing a medium-pressure Hg lamp, as previously described.^33^ AsF_5_ was prepared by static fluorination of As_2_O_3_ in a nickel pressure vessel.^34^

Li_2_PdF_6_ and K_2_PtF_6_ were prepared by oxidation of Pd (Sigma-Aldrich, 99.9%) and Pt (Thermo Scientific, 99.98%) metals with F_2_ in aHF in the presence of LiF (Aldrich, ≥99.98%) and KF (Acros Organics, 99.99%), respectively.^35^

The synthesis of PtF_4_ was conducted in accordance with the previously published procedure, commencing with the oxidation of Pt powder by XeF_2_ in aHF.^36^ The resulting orange-red product^17^ was subsequently decomposed by heating to 430 °C in vacuo, yielding brown PtF_4_, while the released XeF_2_ was collected in a U-shaped FEP trap cooled with liquid nitrogen.^17^

PdF_4_ was prepared by addition of AsF_5_ to Li_2_PdF_6_ partially dissolved in aHF, following a previously reported procedure.^20,37^ However, this reaction was consistently observed to yield Pd_2_F_6_ as a byproduct, which dominated the Raman spectrum of the isolated solid. In an attempt to synthesize high-purity PdF_4_, aHF (2.0 mL) and F_2_ (2.303 mmol) were condensed into a reaction vessel containing a Pd_2_F_6_/PdF_4_ mixture (prepared from 2.293 mmol of Pd), and the vessel was placed, with agitation, under a UV light,^38^ emitted from an air-cooled 1 kW Hg lamp. Following a 39-h period of UV irradiation, the product was isolated and analyzed by Raman spectroscopy. The Raman spectra revealed that the band at 566 cm^–1^, attributable to Pd_2_F_6_, persisted, albeit with markedly reduced intensity. In addition, a very weak band at 1819 cm^–1^ was observed, which is likely indicative of the formation of a fluoridopalladate­(IV) salt containing O_2_^+^ cation. A further 22-h round of UV-aided fluorination was conducted using aHF (2.0 mL) and F_2_ (2.065 mmol), yet the Raman spectrum of the product remained unaltered. Nevertheless, the PXRD of the product revealed only reflections attributable to PdF_4_. This material was subsequently used in the following syntheses.

[XeF]­[AsF_6_] was synthesized by condensing an equimolar amount of AsF_5_ onto a frozen solution of XeF_2_ in aHF.^13^ The product was subsequently isolated by removal of volatiles at −45 °C using a cooled EtOH bath.

XeF_2_·PdF_4_ was synthesized by treatment of PdF_4_ with an excess of molten XeF_2_, followed by removal of the excess XeF_2_ under vacuum at room temperature.^17,21^

### Syntheses

#### Synthesis of [Xe_2_F_3_]­[PtF_3_(XeF_2_)_3_]­[AsF_6_]_2_(*oP*256)

XeF_2_ (52 mg, 0.307 mmol) and PtF_4_ (14 mg, 0.052 mmol) were loaded into an h-shaped FEP vessel, and aHF (1.0 mL) was condensed onto the solids. The mixture was stirred for 20 h using a PTFE-coated magnetic stir bar, yielding a pale-yellow solution above a light-brown powder precipitate. Subsequently, AsF_5_ (0.115 mmol) was condensed into the vessel, resulting in a more intense yellow coloration of the solution, while the light-brown precipitate persisted. Following an additional 2 h of stirring, the solution was decanted into the side arm, and the vessel was placed in a cooling-bath thermostat (Julabo F25-MD or Huber CC2-K6). The main arm was submerged in cooled ethanol, while the side arm containing the solution remained at room temperature. Over the subsequent 13 days, the temperature gradient between the two arms was gradually increased from 2 to 37 °C by lowering the temperature of the cooling bath, causing all aHF to evaporate from the side arm into the main arm and leaving a dark-orange solid material in the side arm. The solvent was then removed from the vessel at room temperature. Upon gentle crushing of the material isolated from the side arm, it was revealed that it consists of colorless and amber-colored crystalline chunks. SCXRD and Raman spectroscopy showed that the amber-colored and colorless crystals corresponded to [Xe_2_F_3_]­[PtF_3_(XeF_2_)_3_]­[AsF_6_]_2_(*oP*256) and [Xe_2_F_3_]­[AsF_6_], respectively. The light-brown material remaining in the main arm exhibited PXRD pattern and Raman spectrum consistent with a mixture of PtF_4_ and [Xe_2_F_3_]­[PtF_3_(XeF_2_)_3_]­[AsF_6_]_2_(*oP*256).

#### Synthesis of [Xe_2_F_3_]­[PtF_3_(XeF_2_)_3_]­[AsF_6_]_2_(*aP*256)

In a typical experiment, [XeF]­[AsF_6_] (49 mg, 0.144 mmol) and K_2_PtF_6_ (21 mg, 0.054 mmol) were loaded into an h-shaped FEP vessel, and aHF (0.8 mL) was dispensed onto the reactants under static vacuum. Initially, a pale-orange solution formed above an orange–red solid. Upon overnight stirring, the orange-red solid converted into a tan-colored, powdered precipitate. The solution was then decanted into the side arm, and the main arm was placed in a cooling bath thermostat. The temperature gradient between the two arms was gradually increased from 4 to 46 °C over a period of 12 days, resulting in the growth of a large colorless crystal of KAsF_6_ surrounded by orange crystalline material of [Xe_2_F_3_]­[PtF_3_(XeF_2_)_3_]­[AsF_6_]_2_(*aP*256), which were analyzed by SCXRD and Raman spectroscopy. The residual tan-colored material in the main arm exhibited no powder diffraction lines and displayed a Raman spectrum matching that reported for *n*XeF_2_·PtF_4_ (1 < *n* < 1.6).^17^

#### Synthesis of [Xe_2_F_3_]­[PdF_3_(XeF_2_)_3_]­[AsF_6_]_2_ from XeF_2_, PdF_4_, and AsF_5_ in aHF

XeF_2_ (73 mg, 0.431 mmol) and PdF_4_ (14 mg, 0.077 mmol) were loaded into an FEP reaction vessel, followed by the addition of aHF (1.0 mL). After stirring for 24 h, most of the brick-red PdF_4_ had dissolved, affording a pale-yellow solution above a small amount of insoluble brown material. Subsequent addition of AsF_5_ (0.189 mmol) produced a solution with a more intense yellow coloration, although some insoluble residue remained at the bottom of the vessel. The clear yellow supernatant was transferred to the side arm, and the main arm of the h-shaped vessel was immersed in a cooling-bath thermostat. The temperature difference between the two arms was increased from 6 to 39 °C over a period of 8 days. The resulting solid orange material in the side arm consisted of colorless crystals of [Xe_2_F_3_]­[AsF_6_] and amber-colored crystals of [Xe_2_F_3_]­[PdF_3_(XeF_2_)_3_]­[AsF_6_]_2_, whereas Raman spectra of the brown solid remaining in the main arm indicated a mixture of XeF_2_·2PdF_4_, [Xe_2_F_3_]­[AsF_6_], PdF_4_, and Pd_2_F_6_.

#### Synthesis of [Xe_2_F_3_]­[PdF_3_(XeF_2_)_3_]­[AsF_6_]_2_ from [XeF]­[AsF_6_], XeF_2_·PdF_4_, and XeF_2_ in aHF

The main arm of an h-shaped FEP vessel was loaded with [XeF]­[AsF_6_] (35 mg, 0.103 mmol), XeF_2_·PdF_4_ (18 mg, 0.051 mmol), XeF_2_ (19 mg, 0.112 mmol), and aHF (1 mL). The mixture was stirred for 90 min, producing a yellow solution and a dark-brown solid precipitate that adhered to the magnetic stirring bar. The solution was decanted into the side arm of the FEP vessel, and the main arm was placed in a cooling-bath thermostat. As the temperature gradient between the arms increased from 5 to 34 °C over the course of 8 days, the solvent gradually evaporated back into the main arm, leaving a solid amber-colored material in the side arm. SCXRD and Raman spectroscopy demonstrated that the amber-colored solid was composed of [Xe_2_F_3_]­[PdF_3_(XeF_2_)_3_]­[AsF_6_]_2_ and [Xe_2_F_3_]­[AsF_6_], whereas Raman spectra of the aHF-insoluble brown material remaining in the main arm indicated that it consisted of XeF_2_·2PdF_4_ and Pd_2_F_6_. The crystal of [Xe_2_F_3_]­[PdF_3_(XeF_2_)_3_]­[AsF_6_]_2_ used for structural characterization by SCXRD was obtained from this preparation.

### Single-Crystal X-Ray Diffraction

#### Crystal Mounting

Crystalline samples were placed on a watch glass and submerged in inert perfluorodecalin oil (ABCR, AB102850, 98%, *cis* and *trans*) inside an inert-atmosphere glovebox. Suitable crystals were selected under polarized light using a stereomicroscope outside the glovebox and attached to a MiTeGen dual-thickness polymer loop. Immediately after the pin was lifted above the surface of the perfluorodecalin, it was grasped with cryo-pin tongs cooled to −196 °C and rapidly transferred to the goniometer head of the diffractometer, where the crystal was protected by a stream of cold nitrogen gas (Oxford Cryosystems 800 Series Cryostream).

#### X-Ray Data Collection, Data Reduction, and Structure Solution

Single-crystal X-ray diffraction data was collected at low temperature (100 K) on a Rigaku OD XtaLAB Synergy-S diffractometer equipped with a Dectris EIGER2 R CdTe 1M hybrid pixel array detector using microfocused Ag *K*α radiation (λ = 0.56087 Å). *CrysAlisPro* software was employed for data processing, utilizing empirical and numerical absorption correction.^39^ The crystal structure of [Xe_2_F_3_]­[PtF_3_(XeF_2_)_3_]­[AsF_6_]_2_(*oP*256) was solved using *SUPERFLIP*,^40^ whereas the crystal structures of [Xe_2_F_3_]­[PtF_3_(XeF_2_)_3_]­[AsF_6_]_2_(*aP*256) and [Xe_2_F_3_]­[PdF_3_(XeF_2_)_3_]­[AsF_6_]_2_ were solved using *SHELXT*.^41^ All three structures were refined with *SHELXL* ^42^ within the *Olex2* software.^43^ Tabulated distances of the nonbonded Xe···F contacts were calculated using the *PLATON* program.^44^ Figures were generated using the *DIAMOND* software.^45^

Because the [Xe_2_F_3_]­[PtF_3_(XeF_2_)_3_]­[AsF_6_]_2_(*oP*256) crystals were always found attached to the colorless byproduct [Xe_2_F_3_]­[AsF_6_], some reflections attributable to the unit cell of the latter were observed after data collection. However, application of a multicrystal data treatment resulted in a structure with inferior agreement parameters. Crystals of [Xe_2_F_3_]­[PtF_3_(XeF_2_)_3_]­[AsF_6_]_2_(*oP*256) and [Xe_2_F_3_]­[PdF_3_(XeF_2_)_3_]­[AsF_6_]_2_ were also found to be twinned. Furthermore, crystals of the *oP*256 and *aP*256 polymorphs of [Xe_2_F_3_]­[PtF_3_(XeF_2_)_3_]­[AsF_6_]_2_ diffracted weakly at high theta angles. Accordingly, the data sets were truncated at resolutions of 0.55 Å and 0.60 Å, respectively.

One symmetry-inequivalent [AsF_6_]^−^ anion in the crystal structure of [Xe_2_F_3_]­[PtF_3_(XeF_2_)_3_]­[AsF_6_]_2_(*oP*256) exhibited disorder, which was treated by splitting four axial atoms and applying SADI and RIGU restraints during refinement.

### Vibrational Spectroscopy

#### Raman Spectroscopy

Raman spectra were measured at 100 K using a Bruker Senterra II confocal Raman microscope equipped with a Linkam LTS420 low-temperature stage. The samples were excited using a 785 nm laser at either 25 mW in the case of [Xe_2_F_3_]­[PtF_3_(XeF_2_)_3_]­[AsF_6_]_2_(*oP*256) or 50 mW in the case of [Xe_2_F_3_]­[PtF_3_(XeF_2_)_3_]­[AsF_6_]_2_(*aP*256) and [Xe_2_F_3_]­[PdF_3_(XeF_2_)_3_]­[AsF_6_]_2_. Spectra were measured over the 50–1410 cm^–1^ range with a spectral resolution of 1.5 cm^–1^ using a 50 μm aperture. Samples were prepared by coarsely homogenizing the reaction products in an agate mortar inside a glovebox and loading the material into quartz capillaries, which had been thoroughly dried and passivated with elemental F_2_ beforehand. Crystals of the [MF_3_(XeF_2_)_3_]^+^ salts invariably formed in the presence of [Xe_2_F_3_]­[AsF_6_] and KAsF_6_ byproducts and could not be readily separated. Consequently, Raman spectra of the target compounds were obtained from isolated, brightly colored crystalline particulates that could be visually distinguished from the colorless [Xe_2_F_3_]­[AsF_6_] or KAsF_6_ crystals. The plotted spectra (Figure 5) were obtained by averaging spectra from multiple, distinct, randomly oriented crystallites. The number of averaged spectra was 7 for both the *oP*256 and *aP*256 polymorphs of [Xe_2_F_3_]­[PtF_3_(XeF_2_)_3_]­[AsF_6_]_2_ and 13 for [Xe_2_F_3_]­[PdF_3_(XeF_2_)_3_]­[AsF_6_]_2_.

#### Infrared Spectroscopy

ATR-IR spectra were measured on a Bruker Alpha II FT-IR spectrometer equipped with a Platinum Diamond-ATR sampling module, operated inside an N_2_-atmosphere glovebox. Spectra were measured by accumulation of 24 scans over the 400–4000 cm^–1^ range at a resolution of 4 cm^–1^. The spectra were obtained from finely pulverized materials, which consisted of mixtures of the [MF_3_(XeF_2_)_3_]^+^ salts and [Xe_2_F_3_]­[AsF_6_] or KAsF_6_ byproducts (Figure S12). For comparison, ATR-IR spectra of pure [Xe_2_F_3_]­[AsF_6_] and KAsF_6_ were also recorded under identical conditions (Figure S13).

### Computational Details

Quantum-chemical calculations were carried out using density functional theory (DFT) as implemented in the *ORCA* software package (version 6.0.0).^25,46,47^ The electronic structures of the [MF_3_(XeF_2_)_3_]^+^ cations were calculated starting from the crystallographic coordinates and converged to stationary points with all vibrational frequencies real. The calculations were performed using the PBE0-D3 functional,^48–51^ aug-cc-pVQZ for F, and aug-cc-pVQZ-PP basis sets with SK-MCDHF-RSC effective core potentials for the heavy atoms Pd,^52^ Pt,^53^ and Xe.^54^

Raman spectra were calculated using numerical frequency calculations (!NumFreq) together with polarizability calculations (%elprop Polar 1 end).^55^ Raman intensities were calculated using the *ChemCraft* ^56^ Raman spectra utility with a temperature of 298.15 K and incident laser frequency of 10,000 cm^–1^. Gaussian broadening with a full width at half-maximum of 2 cm^–1^ was applied.

Computational results were processed using the *Multiwfn* software^57,58^ (version 3.8­(dev)) to perform Mayer bond order analysis,^59,60^ and quantitative molecular surface analyses.^27,61,62^ *GaussView* ^63^ was used to visualize the MEPS.

## Supplementary Material



## Data Availability

The data supporting the findings of this study are available in the Zenodo open repository (https://zenodo.org/) under the DOI number: 10.5281/zenodo.17787891.

## Notes

Additional crystallographic data and figures, DFT-calculated vibrational frequencies and structural parameters of the [MF_3_(XeF_2_)_3_]^+^ cations and their comparison with experimental values, a table of observed Raman band positions and their relative intensities, and ATR-IR spectra (PDF)

## Notes

In memory of Professor Dr. Boris Frlec (1936–2026).
