# Supplementary material for: Coordination of XeF2 to Fluoridometal Cations: The Adduct Cations [PtF3(XeF2)3]+ and [PdF3(XeF2)3]+
Source: Inorg Chem. 2026 Feb 26;65(10):5668–77. doi: 10.1021/acs.inorgchem.5c05940 (PMC12997165; doi:10.1021/acs.inorgchem.5c05940)
Supplement: Supplementary file 1 [file ic5c05940_si_001.pdf]

Supporting Information

# Coordination of $XeF_2$ to Fluoridometal Cations: The Adduct Cations $[PtF_3(XeF_2)_3]^+$ and $[PdF_3(XeF_2)_3]^+$

*Klemen Motaln,*[1,2] *Miha Virant,*[1] *Matic Lozinšek*[1,2,*]

[1] *Jožef Stefan Institute, Jamova cesta 39, 1000 Ljubljana, Slovenia*

[2] *Jožef Stefan International Postgraduate School, Jamova cesta 39, 1000 Ljubljana, Slovenia*

**Corresponding authors' e-mail: matic.lozinsek@ijs.si*

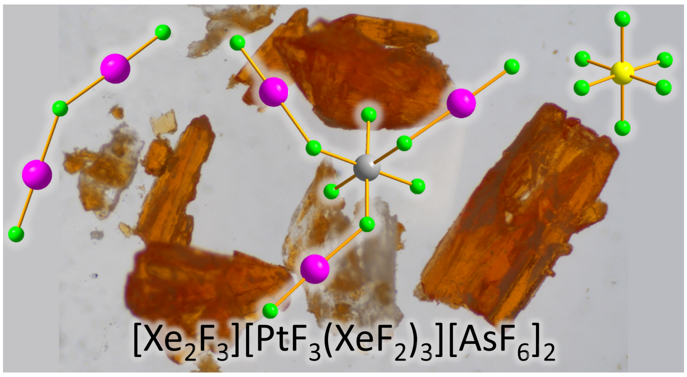


DOI: 10.1021/acs.inorgchem.5c05940

# Table of Contents

**Figure S1.** Amber-colored crystals of $[Xe_2F_3][PtF_3(XeF_2)_3][AsF_6]_2$(*aP*256) and $[Xe_2F_3][PdF_3(XeF_2)_3][AsF_6]_2$ ............ S3

**Table S1.** Summary of crystallographic data and refinement results. ............ S4

**Table S2.** Experimental (SCXRD) geometrical parameters for $[Xe_2F_3][PtF_3(XeF_2)_3][AsF_6]_2$(*oP*256). ............ S5

**Table S3.** Experimental (SCXRD) geometrical parameters for $[Xe_2F_3][PtF_3(XeF_2)_3][AsF_6]_2$(*aP*256). ............ S7

**Table S4.** Experimental (SCXRD) geometrical parameters for $[Xe_2F_3][PdF_3(XeF_2)_3][AsF_6]_2$. ............ S10

**Figure S2.** Composite view of the asymmetric unit (*Z′* = 2) of $[Xe_2F_3][PtF_3(XeF_2)_3][AsF_6]_2$(*oP*256); view of the asymmetric unit (*Z′* = 1) of $[Xe_2F_3][PdF_3(XeF_2)_3][AsF_6]_2$ ............ S11

**Figure S3.** Composite view of the asymmetric unit (*Z′* = 4) of $[Xe_2F_3][PtF_3(XeF_2)_3][AsF_6]_2$(*aP*256) ............ S12

**Figure S4.** The crystal packing and the unit cell of $[Xe_2F_3][PtF_3(XeF_2)_3][AsF_6]_2$(*oP*256) ............ S13

**Figure S5.** The crystal packing and the unit cell of $[Xe_2F_3][PtF_3(XeF_2)_3][AsF_6]_2$(*aP*256) ............ S14

**Figure S6.** The crystal packing and the unit cell of $[Xe_2F_3][PdF_3(XeF_2)_3][AsF_6]_2$ ............ S15

**Table S5.** Non-bonded inter- and intramolecular Xe···F contacts in $[Xe_2F_3][PtF_3(XeF_2)_3][AsF_6]_2$(*oP*256) ............ S16

**Table S6.** Non-bonded inter- and intramolecular Xe···F contacts in $[Xe_2F_3][PtF_3(XeF_2)_3][AsF_6]_2$(*aP*256) ............ S17

**Table S7.** Non-bonded inter- and intramolecular Xe···F contacts in $[Xe_2F_3][PdF_3(XeF_2)_3][AsF_6]_2$ ............ S17

**Figure S7.** Overlay of crystallographically independent $[PtF_3(XeF_2)_3]^+$ adduct cations ............ S18

**Table S8.** Calculated vibrational frequencies of the $[MF_3(XeF_2)_3]^+$ cations ............ S19

**Table S9.** Optimized geometry of $[PtF_3(XeF_2)_3]^+$ cation ............ S26

**Table S10.** Optimized geometry of $[PdF_3(XeF_2)_3]^+$ cation ............ S26

**Table S11.** Calculated geometrical parameters of the $[PtF_3(XeF_2)_3]^+$ cation. ............ S27

**Table S12.** Calculated geometrical parameters of the $[PdF_3(XeF_2)_3]^+$ cation ............ S28

**Table S13.** Calculated AIM charges and Mayer valences for the $[MF_3(XeF_2)_3]^+$ cations and free $XeF_2$. ............ S29

**Figure S8.** MEPS of $[MF_3]^+$ cations after removing the $XeF_2$ units ............ S30

**Figure S9.** MEPS of the $[MF_3]^+$ cations with optimized geometries ............ S30

**Table S14.** Optimized geometries of $[MF_3]^+$ cations ............ S31

**Table S15.** Optimized geometries of $[MF_6]^{2-}$ anions ............ S31

**Table S16.** Calculated AIM charges, Mayer valences, and Mayer bond orders for the $[MF_6]^{2-}$ anions. ............ S31

**Table S17.** QTAIM topological analysis of $[MF_3(XeF_2)_3]^+$ cations and free $XeF_2$ ............ S32

**Table S18.** Observed bands in the low-temperature Raman spectra of $[Xe_2F_3][PtF_3(XeF_2)_3][AsF_6]_2$(*oP*256), $[Xe_2F_3][PtF_3(XeF_2)_3][AsF_6]_2$(*aP*256), and $[Xe_2F_3][PdF_3(XeF_2)_3][AsF_6]_2$ ............ S33

**Figure S10.** Comparison of the calculated Raman spectrum of $[PtF_3(XeF_2)_3]^+$ cation and experimentally measured Raman spectra of the two polymorphs of $[Xe_2F_3][PtF_3(XeF_2)_3][AsF_6]_2$ and $[Xe_2F_3][AsF_6]$ ............ S34

**Figure S11.** Comparison of the calculated Raman spectrum of $[PdF_3(XeF_2)_3]^+$ cation and experimentally measured Raman spectra of $[Xe_2F_3][PdF_3(XeF_2)_3][AsF_6]_2$ and $[Xe_2F_3][AsF_6]$ ............ S35

**Figure S12.** ATR-IR spectra recorded on powdered samples containing $[Xe_2F_3][PdF_3(XeF_2)_3][AsF_6]_2$(*oP*256), $[Xe_2F_3][PtF_3(XeF_2)_3][AsF_6]_2$(*aP*256), $[Xe_2F_3][PdF_3(XeF_2)_3][AsF_6]_2$, as well as $[Xe_2F_3][AsF_6]$ and $KAsF_6$ impurities. ... S36

**Figure S13.** ATR-IR spectra of $[Xe_2F_3][AsF_6]$ and $KAsF_6$ ............ S36

References ............ S37

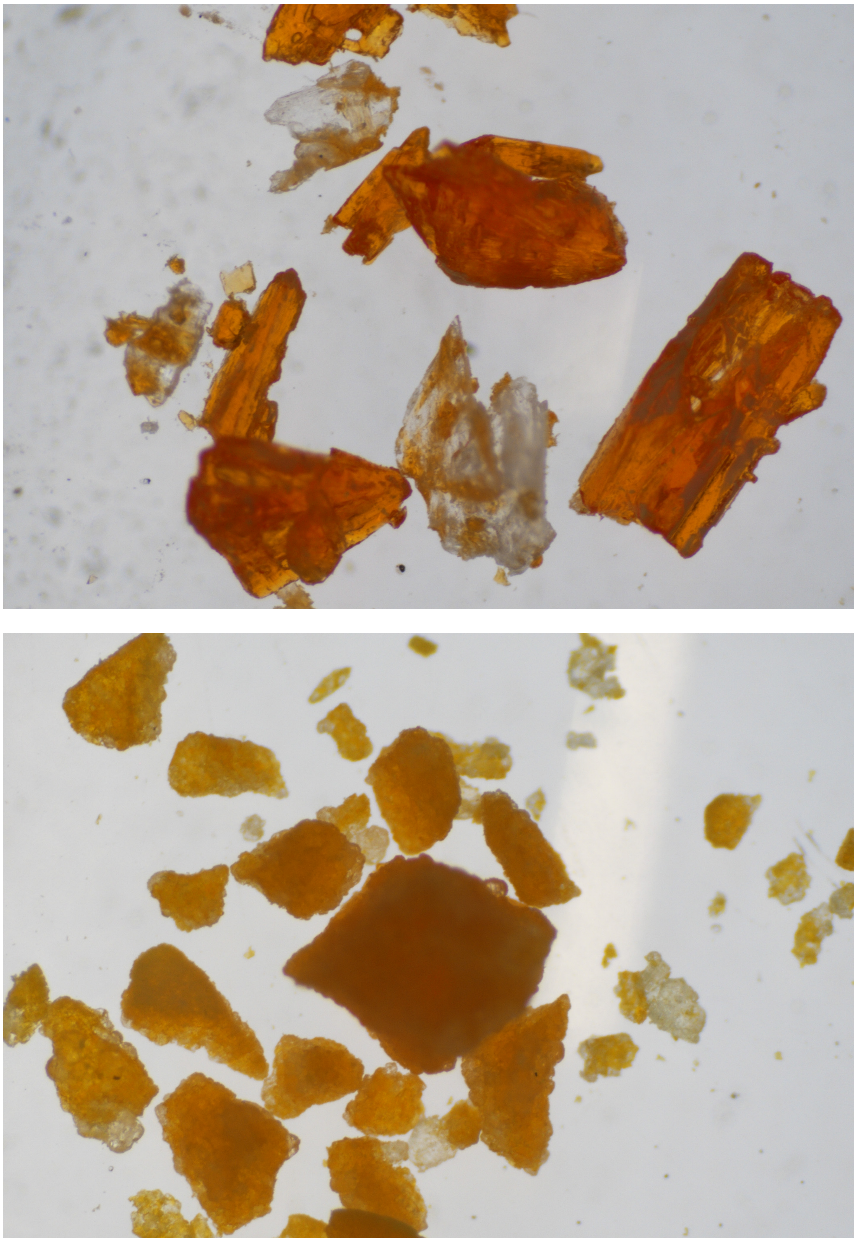

**Figure S1.** Amber-colored crystals of $[Xe_2F_3][PtF_3(XeF_2)_3][AsF_6]_2$($aP$256) (top) and $[Xe_2F_3][PdF_3(XeF_2)_3][AsF_6]_2$ (bottom), viewed under a stereomicroscope at 30× magnification. The colorless crystals admixed with these phases are $KAsF_6$ (top) and $[Xe_2F_3][AsF_6]$ (bottom), respectively.

**Table S1.** Summary of crystallographic data and refinement results.

| Compound | $[Xe_2F_3][PtF_3(XeF_2)_3][AsF_6]_2$(*oP*256)[a] | $[Xe_2F_3][PtF_3(XeF_2)_3][AsF_6]_2$(*aP*256) | $[Xe_2F_3][PdF_3(XeF_2)_3][AsF_6]_2$[b] |
|---|---|---|---|
| Formula | $Xe_5PtAs_2F_{24}$ | $Xe_5PtAs_2F_{24}$ | $Xe_5PdAs_2F_{24}$ |
| $F_w$ | 1457.43 | 1457.43 | 1368.74 |
| $T$ (K) | 100.00(10) | 100.00(10) | 100.00(10) |
| Crystal system | Orthorhombic | Triclinic | Monoclinic |
| Space group | $P2_12_12_1$ | $P\bar{1}$ | $P2_1$ |
| $a$ (Å) | 11.86164(10) | 12.92922(7) | 6.53000(4) |
| $b$ (Å) | 16.48399(13) | 13.07591(6) | 11.83284(6) |
| $c$ (Å) | 23.8108(2) | 27.91602(13) | 14.93053(9) |
| α (°) | 90 | 83.6237(4) | 90 |
| β (°) | 90 | 79.6369(4) | 90.0233(5) |
| γ (°) | 90 | 75.7149(5) | 90 |
| $V$ (Å$^3$) | 4655.66(7) | 4487.97(4) | 1153.658(11) |
| $Z$ | 8 | 8 | 2 |
| $\rho_{calc}$ (g/cm$^3$) | 4.159 | 4.314 | 3.940 |
| Crystal size (mm) | 0.155 × 0.097 × 0.053 | 0.373 × 0.145 × 0.136 | 0.272 × 0.243 × 0.165 |
| Radiation type | Ag *K*α | Ag *K*α | Ag *K*α |
| λ (Å) | 0.56087 | 0.56087 | 0.56087 |
| μ (mm$^{-1}$) | 8.639 | 8.962 | 5.838 |
| $F$(000) | 5040 | 5040 | 1196 |
| $\theta_{max}$ (°) | 30.656[c] | 27.865[d] | 36.876 |
| Index ranges | $-21 \le h \le 21$ | $-21 \le h \le 21$ | $-13 \le h \le 13$ |
| | $-29 \le k \le 29$ | $-21 \le k \le 21$ | $-24 \le k \le 25$ |
| | $-43 \le l \le 43$ | $-46 \le l \le 46$ | $-31 \le l \le 31$ |
| Reflections collected | 348037 | 528951 | 246799 |
| Independent reflections | 29267 | 43545 | 22248 |
| Reflections with [$I > 2\sigma(I)$] | 26495 | 40558 | 21189 |
| $R_{int}$ | 0.1229 | 0.0970 | 0.0969 |
| $R_{sigma}$ | 0.0416 | 0.0295 | 0.0327 |
| Data/restraints/parameters | 29267/56/615 | 43545/0/1153 | 22248/1/290 |
| $S$ | 1.033 | 1.173 | 1.033 |
| $R_1$, $wR_2$ [$I > 2\sigma(I)$] | 0.0389, 0.0983 | 0.0319, 0.0802 | 0.0320, 0.0782 |
| $R_1$, $wR_2$ [all data] | 0.0433, 0.1001 | 0.0344, 0.0811 | 0.0343, 0.0797 |
| $\Delta\rho_{min}$, $\Delta\rho_{max}$ (e/Å$^3$) | −1.958, 5.733 | −2.818, 4.386 | −1.959, 1.990 |
| CSD deposition number[e] | 2517119 | 2517120 | 2517118 |

[a] Refined as a two-component inversion twin with twin ratio of 0.694:0.306.

[b] Refined as a two-component twin [1 0 0 0 1 0 0 0 −1] with twin ratio of 0.5244(7):0.4756(7).

[c] Dataset truncated to 0.55 Å resolution during data reduction in *CrysAlisPro* software.

[d] Dataset truncated to 0.60 Å resolution during data reduction in *CrysAlisPro* software.

[e] CSD 2517118–2517120 contain the supplementary crystallographic data for this paper. These data can be obtained free of charge from FIZ Karlsruhe via https://www.ccdc.cam.ac.uk/structures.

**Table S2.** Experimental (SCXRD) geometrical parameters for $[Xe_2F_3][PtF_3(XeF_2)_3][AsF_6]_2$(*oP*256).

| | | | | | |
|---|---|---|---|---|---|
| **Bond distances** (Å) | | | | | |
| Xe1—F1 | 1.906(5) | Pt1—F8 | 1.978(5) | As1—F29B | 1.733(13) |
| Xe1—F2 | 2.163(5) | Pt1—F10 | 1.980(5) | As1—F28B | 1.786(13) |
| Xe2—F2 | 2.125(5) | Pt1—F12 | 1.996(5) | As2—F31 | 1.699(6) |
| Xe2—F3 | 1.921(5) | Pt1—F13 | 1.881(5) | As2—F32 | 1.713(5) |
| Xe3—F4 | 1.912(5) | Pt1—F14 | 1.888(5) | As2—F33 | 1.713(5) |
| Xe3—F5 | 2.174(5) | Pt1—F15 | 1.895(5) | As2—F34 | 1.723(6) |
| Xe4—F5 | 2.128(5) | Pt2—F17 | 1.991(4) | As2—F35 | 1.724(6) |
| Xe4—F6 | 1.929(5) | Pt2—F19 | 1.986(5) | As2—F36 | 1.729(7) |
| Xe5—F7 | 1.888(6) | Pt2—F21 | 1.984(5) | As3—F37 | 1.702(7) |
| Xe5—F8 | 2.207(5) | Pt2—F22 | 1.889(5) | As3—F38 | 1.704(7) |
| Xe6—F9 | 1.912(6) | Pt2—F23 | 1.889(5) | As3—F39 | 1.707(6) |
| Xe6—F10 | 2.186(5) | Pt2—F24 | 1.898(5) | As3—F40 | 1.720(6) |
| Xe7—F11 | 1.918(5) | As1—F25 | 1.685(8) | As3—F41 | 1.724(6) |
| Xe7—F12 | 2.184(5) | As1—F26 | 1.732(7) | As3—F42 | 1.740(6) |
| Xe8—F16 | 1.903(6) | As1—F27A | 1.683(12) | As4—F43 | 1.705(6) |
| Xe8—F17 | 2.211(5) | As1—F28A | 1.681(11) | As4—F44 | 1.705(7) |
| Xe9—F18 | 1.907(5) | As1—F29A | 1.727(12) | As4—F45 | 1.714(6) |
| Xe9—F19 | 2.182(5) | As1—F30A | 1.751(10) | As4—F46 | 1.726(5) |
| Xe10—F20 | 1.921(6) | As1—F27B | 1.705(16) | As4—F47 | 1.728(5) |
| Xe10—F21 | 2.206(5) | As1—F30B | 1.657(13) | As4—F48 | 1.737(5) |
| **Angles** (°) | | | | | |
| F1—Xe1—F2 | 177.1(3) | F23—Pt2—F21 | 178.9(2) | F33—As2—F34 | 90.9(3) |
| F3—Xe2—F2 | 177.6(2) | F23—Pt2—F24 | 92.8(2) | F33—As2—F35 | 90.6(3) |
| Xe2—F2—Xe1 | 140.3(3) | F24—Pt2—F17 | 89.2(2) | F33—As2—F36 | 89.6(3) |
| F4—Xe3—F5 | 178.9(3) | F24—Pt2—F19 | 178.8(2) | F34—As2—F35 | 91.2(4) |
| F6—Xe4—F5 | 178.7(2) | F24—Pt2—F21 | 88.1(2) | F34—As2—F36 | 178.7(4) |
| Xe4—F5—Xe3 | 139.9(3) | F25—As1—F26 | 177.8(4) | F35—As2—F36 | 87.5(4) |
| F7—Xe5—F8 | 176.4(3) | F25—As1—F29A | 94.3(5) | F37—As3—F38 | 93.2(5) |
| F9—Xe6—F10 | 176.5(3) | F25—As1—F30A | 92.1(6) | F37—As3—F39 | 90.7(4) |
| F11—Xe7—F12 | 176.8(2) | F25—As1—F27B | 89.0(9) | F37—As3—F40 | 89.9(4) |
| F16—Xe8—F17 | 176.4(3) | F25—As1—F29B | 82.0(7) | F37—As3—F41 | 90.1(4) |
| F18—Xe9—F19 | 175.9(2) | F25—As1—F28B | 91.3(6) | F37—As3—F42 | 179.1(4) |
| F20—Xe10—F21 | 176.0(2) | F26—As1—F30A | 86.6(5) | F38—As3—F39 | 90.3(5) |
| Pt1—F8—Xe5 | 123.0(2) | F26—As1—F29B | 97.1(7) | F38—As3—F40 | 90.1(4) |
| Pt1—F10—Xe6 | 125.7(3) | F26—As1—F28B | 90.7(5) | F38—As3—F41 | 176.7(5) |
| Pt1—F12—Xe7 | 121.2(2) | F27A—As1—F25 | 89.1(8) | F38—As3—F42 | 87.7(5) |
| Pt2—F17—Xe8 | 126.0(2) | F27A—As1—F26 | 92.7(8) | F39—As3—F40 | 179.3(4) |

**Table S2**. Continued.

| Angles (°) | | | | | |
|---|---|---|---|---|---|
| Pt2—F19—Xe9 | 127.4(2) | F27A—As1—F29A | 175.7(8) | F39—As3—F41 | 89.3(4) |
| Pt2—F21—Xe10 | 121.7(2) | F27A—As1—F30A | 88.2(8) | F39—As3—F42 | 89.1(3) |
| F8—Pt1—F10 | 91.2(2) | F28A—As1—F25 | 93.1(6) | F40—As3—F41 | 90.2(4) |
| F8—Pt1—F12 | 92.0(2) | F28A—As1—F26 | 88.2(6) | F40—As3—F42 | 90.3(3) |
| F10—Pt1—F12 | 92.3(2) | F28A—As1—F27A | 91.5(9) | F41—As3—F42 | 89.1(3) |
| F13—Pt1—F8 | 178.5(2) | F28A—As1—F29A | 90.9(9) | F43—As4—F45 | 91.9(3) |
| F13—Pt1—F10 | 87.8(2) | F28A—As1—F30A | 174.8(7) | F43—As4—F46 | 90.1(4) |
| F13—Pt1—F12 | 87.0(2) | F29A—As1—F26 | 83.9(6) | F43—As4—F47 | 90.1(3) |
| F13—Pt1—F14 | 92.8(2) | F29A—As1—F30A | 89.1(7) | F43—As4—F48 | 178.2(3) |
| F13—Pt1—F15 | 93.3(2) | F27B—As1—F26 | 92.1(9) | F44—As4—F43 | 91.6(4) |
| F14—Pt1—F8 | 88.3(2) | F27B—As1—F29B | 169.0(11) | F44—As4—F45 | 90.6(4) |
| F14—Pt1—F10 | 178.3(2) | F27B—As1—F28B | 87.6(9) | F44—As4—F46 | 178.1(4) |
| F14—Pt1—F12 | 89.3(2) | F30B—As1—F25 | 79.7(8) | F44—As4—F47 | 90.2(3) |
| F14—Pt1—F15 | 90.4(2) | F30B—As1—F26 | 98.3(8) | F44—As4—F48 | 89.0(4) |
| F15—Pt1—F8 | 87.7(2) | F30B—As1—F27B | 91.5(11) | F45—As4—F46 | 89.8(3) |
| F15—Pt1—F10 | 87.9(2) | F30B—As1—F29B | 93.1(10) | F45—As4—F47 | 177.8(3) |
| F15—Pt1—F12 | 179.6(2) | F30B—As1—F28B | 171.0(9) | F45—As4—F48 | 89.8(3) |
| F19—Pt2—F17 | 90.0(2) | F29B—As1—F28B | 86.3(9) | F46—As4—F47 | 89.2(3) |
| F21—Pt2—F17 | 90.3(2) | F31—As2—F32 | 90.9(4) | F46—As4—F48 | 89.2(3) |
| F21—Pt2—F19 | 91.0(2) | F31—As2—F33 | 89.7(4) | F47—As4—F48 | 88.2(3) |
| F22—Pt2—F17 | 179.0(2) | F31—As2—F34 | 90.8(4) | Xe5—F8—Pt1—F14 | −38.4 |
| F22—Pt2—F19 | 89.9(2) | F31—As2—F35 | 177.9(4) | Xe6—F10—Pt1—F13 | −31.8(3) |
| F22—Pt2—F21 | 88.7(2) | F31—As2—F36 | 90.4(4) | Xe7—F12—Pt1—F14 | 0.1(3) |
| F22—Pt2—F23 | 91.9(3) | F32—As2—F33 | 179.0(3) | Xe8—F17—Pt2—F23 | −24.9(3) |
| F22—Pt2—F24 | 90.9(2) | F32—As2—F34 | 90.0(3) | Xe9—F19—Pt2—F22 | 17.1(4) |
| F23—Pt2—F17 | 89.1(2) | F32—As2—F35 | 88.8(3) | Xe10—F21—Pt2—F22 | −37.0(4) |
| F23—Pt2—F19 | 88.1(2) | F32—As2—F36 | 89.5(3) | | |

**Table S3.** Experimental (SCXRD) geometrical parameters for $[Xe_2F_3][PtF_3(XeF_2)_3][AsF_6]_2$(*aP*256).

| Bond distances (Å) | | | | | |
|---|---|---|---|---|---|
| Xe1—F1 | 1.908(3) | Xe20—F44 | 1.918(3) | As2—F60 | 1.728(3) |
| Xe1—F2 | 2.157(3) | Xe20—F45 | 2.182(3) | As3—F61 | 1.706(3) |
| Xe2—F2 | 2.173(3) | Pt1—F14 | 1.981(3) | As3—F62 | 1.714(3) |
| Xe2—F3 | 1.910(3) | Pt1—F16 | 1.996(3) | As3—F63 | 1.717(3) |
| Xe3—F4 | 1.910(3) | Pt1—F18 | 1.992(3) | As3—F64 | 1.724(3) |
| Xe3—F5 | 2.188(3) | Pt1—F19 | 1.882(3) | As3—F65 | 1.727(3) |
| Xe4—F5 | 2.169(3) | Pt1—F20 | 1.886(3) | As3—F66 | 1.731(3) |
| Xe4—F6 | 1.919(3) | Pt1—F21 | 1.892(3) | As4—F67 | 1.707(3) |
| Xe5—F7 | 1.918(3) | Pt2—F23 | 1.983(3) | As4—F68 | 1.710(3) |
| Xe5—F8 | 2.176(3) | Pt2—F25 | 1.993(2) | As4—F69 | 1.714(3) |
| Xe6—F8 | 2.158(3) | Pt2—F27 | 2.002(2) | As4—F70 | 1.718(3) |
| Xe6—F9 | 1.922(3) | Pt2—F28 | 1.886(3) | As4—F71 | 1.726(3) |
| Xe7—F10 | 1.921(3) | Pt2—F29 | 1.889(3) | As4—F72 | 1.734(3) |
| Xe7—F11 | 2.164(3) | Pt2—F30 | 1.897(2) | As5—F73 | 1.709(3) |
| Xe8—F11 | 2.180(3) | Pt3—F32 | 1.985(2) | As5—F74 | 1.720(3) |
| Xe8—F12 | 1.923(3) | Pt3—F34 | 1.992(2) | As5—F75 | 1.725(3) |
| Xe9—F13 | 1.898(3) | Pt3—F36 | 1.991(2) | As5—F76 | 1.725(3) |
| Xe9—F14 | 2.206(3) | Pt3—F37 | 1.886(2) | As5—F77 | 1.725(3) |
| Xe10—F15 | 1.905(3) | Pt3—F38 | 1.891(2) | As5—F78 | 1.732(3) |
| Xe10—F16 | 2.203(3) | Pt3—F39 | 1.891(3) | As6—F79 | 1.710(3) |
| Xe11—F17 | 1.909(3) | Pt4—F41 | 1.987(3) | As6—F80 | 1.716(3) |
| Xe11—F18 | 2.206(3) | Pt4—F43 | 1.983(3) | As6—F81 | 1.717(3) |
| Xe12—F22 | 1.901(3) | Pt4—F45 | 1.992(3) | As6—F82 | 1.718(3) |
| Xe12—F23 | 2.203(3) | Pt4—F46 | 1.883(3) | As6—F83 | 1.723(3) |
| Xe13—F24 | 1.906(3) | Pt4—F47 | 1.884(3) | As6—F84 | 1.731(3) |
| Xe13—F25 | 2.202(3) | Pt4—F48 | 1.888(3) | As7—F85 | 1.712(3) |
| Xe14—F26 | 1.914(3) | As1—F49 | 1.704(3) | As7—F86 | 1.715(3) |
| Xe14—F27 | 2.194(3) | As1—F50 | 1.707(3) | As7—F87 | 1.724(3) |
| Xe15—F31 | 1.904(3) | As1—F51 | 1.717(3) | As7—F88 | 1.724(3) |
| Xe15—F32 | 2.207(3) | As1—F52 | 1.722(3) | As7—F89 | 1.726(3) |
| Xe16—F33 | 1.909(3) | As1—F53 | 1.725(3) | As7—F90 | 1.731(3) |
| Xe16—F34 | 2.214(2) | As1—F54 | 1.737(3) | As8—F91 | 1.714(3) |
| Xe17—F35 | 1.911(3) | As2—F55 | 1.705(3) | As8—F92 | 1.715(3) |
| Xe17—F36 | 2.208(2) | As2—F56 | 1.718(4) | As8—F93 | 1.715(3) |
| Xe18—F40 | 1.906(3) | As2—F57 | 1.719(3) | As8—F94 | 1.719(3) |
| Xe18—F41 | 2.194(3) | As2—F58 | 1.719(3) | As8—F95 | 1.721(3) |
| Xe19—F42 | 1.911(3) | As2—F59 | 1.722(3) | As8—F96 | 1.728(3) |
| Xe19—F43 | 2.179(3) | | | | |

**Table S3.** Continued.

| Angles (°) | | | | | |
|---|---|---|---|---|---|
| F1—Xe1—F2 | 178.53(17) | F38—Pt3—F36 | 91.84(11) | F69—As4—F72 | 89.64(16) |
| F3—Xe2—F2 | 178.54(14) | F39—Pt3—F32 | 89.02(12) | F70—As4—F71 | 90.81(18) |
| Xe1—F2—Xe2 | 134.65(15) | F39—Pt3—F34 | 178.36(12) | F70—As4—F72 | 179.28(18) |
| F4—Xe3—F5 | 178.22(13) | F39—Pt3—F36 | 89.39(11) | F71—As4—F72 | 88.46(15) |
| F6—Xe4—F5 | 179.36(14) | F39—Pt3—F38 | 92.53(12) | F73—As5—F74 | 91.72(15) |
| Xe4—F5—Xe3 | 127.10(13) | F41—Pt4—F45 | 88.63(11) | F73—As5—F75 | 90.31(16) |
| F7—Xe5—F8 | 179.18(15) | F43—Pt4—F41 | 85.56(12) | F73—As5—F76 | 177.95(15) |
| F9—Xe6—F8 | 178.11(15) | F43—Pt4—F45 | 90.30(12) | F73—As5—F77 | 90.09(15) |
| Xe6—F8—Xe5 | 129.36(15) | F46—Pt4—F41 | 90.41(13) | F73—As5—F78 | 89.23(14) |
| F10—Xe7—F11 | 178.16(13) | F46—Pt4—F43 | 90.51(14) | F74—As5—F75 | 89.82(14) |
| F12—Xe8—F11 | 178.90(12) | F46—Pt4—F45 | 178.69(14) | F74—As5—F76 | 89.89(14) |
| Xe7—F11—Xe8 | 134.54(14) | F46—Pt4—F47 | 91.30(14) | F74—As5—F77 | 178.04(15) |
| F13—Xe9—F14 | 175.96(14) | F46—Pt4—F48 | 91.23(15) | F74—As5—F78 | 89.26(14) |
| F15—Xe10—F16 | 173.35(14) | F47—Pt4—F41 | 177.32(13) | F75—As5—F76 | 90.97(15) |
| F17—Xe11—F18 | 178.44(13) | F47—Pt4—F43 | 92.36(13) | F75—As5—F78 | 178.96(14) |
| F22—Xe12—F23 | 174.30(12) | F47—Pt4—F45 | 89.69(13) | F76—As5—F78 | 89.51(14) |
| F24—Xe13—F25 | 179.35(12) | F47—Pt4—F48 | 89.90(15) | F77—As5—F75 | 90.94(15) |
| F26—Xe14—F27 | 178.67(12) | F48—Pt4—F41 | 92.12(13) | F77—As5—F76 | 88.29(14) |
| F31—Xe15—F32 | 177.34(13) | F48—Pt4—F43 | 177.12(12) | F77—As5—F78 | 89.99(14) |
| F33—Xe16—F34 | 174.79(11) | F48—Pt4—F45 | 87.92(14) | F79—As6—F80 | 90.69(19) |
| F35—Xe17—F36 | 179.40(12) | F49—As1—F50 | 91.2(2) | F79—As6—F81 | 178.23(18) |
| F40—Xe18—F41 | 177.86(13) | F49—As1—F51 | 90.59(19) | F79—As6—F82 | 89.91(17) |
| F42—Xe19—F43 | 177.14(13) | F49—As1—F52 | 178.68(19) | F79—As6—F83 | 89.99(17) |
| F44—Xe20—F45 | 177.51(12) | F49—As1—F53 | 89.71(16) | F79—As6—F84 | 89.09(15) |
| Pt1—F14—Xe9 | 130.90(15) | F49—As1—F54 | 89.78(16) | F80—As6—F81 | 90.82(19) |
| Pt1—F16—Xe10 | 142.27(16) | F50—As1—F51 | 178.16(18) | F80—As6—F82 | 90.25(17) |
| Pt1—F18—Xe11 | 124.49(13) | F50—As1—F52 | 90.13(17) | F80—As6—F83 | 90.23(17) |
| Pt2—F23—Xe12 | 136.03(15) | F50—As1—F53 | 90.17(15) | F80—As6—F84 | 179.73(17) |
| Pt2—F25—Xe13 | 123.77(12) | F50—As1—F54 | 89.36(15) | F81—As6—F82 | 89.18(18) |
| Pt2—F27—Xe14 | 129.56(13) | F51—As1—F52 | 88.11(17) | F81—As6—F83 | 90.91(18) |
| Pt3—F32—Xe15 | 128.03(13) | F51—As1—F53 | 90.35(16) | F81—As6—F84 | 89.40(16) |
| Pt3—F34—Xe16 | 129.60(12) | F51—As1—F54 | 90.14(16) | F82—As6—F83 | 179.52(18) |
| Pt3—F36—Xe17 | 126.33(12) | F52—As1—F53 | 90.50(15) | F82—As6—F84 | 89.92(16) |
| Pt4—F41—Xe18 | 129.32(13) | F52—As1—F54 | 90.03(15) | F83—As6—F84 | 89.61(16) |
| Pt4—F43—Xe19 | 132.35(15) | F53—As1—F54 | 179.29(15) | F85—As7—F86 | 91.48(16) |
| Pt4—F45—Xe20 | 126.82(13) | F55—As2—F56 | 91.0(2) | F85—As7—F87 | 90.29(15) |
| F14—Pt1—F16 | 90.06(15) | F55—As2—F57 | 90.35(19) | F85—As7—F88 | 178.27(15) |
| F14—Pt1—F18 | 88.69(12) | F55—As2—F58 | 89.72(17) | F85—As7—F89 | 89.64(16) |

**Table S3.** Continued.

| Angles (°) | | | | | |
|---|---|---|---|---|---|
| F18—Pt1—F16 | 88.44(12) | F55—As2—F59 | 90.94(16) | F85—As7—F90 | 90.20(14) |
| F19—Pt1—F14 | 89.61(16) | F55—As2—F60 | 179.16(18) | F86—As7—F87 | 90.14(15) |
| F19—Pt1—F16 | 178.24(13) | F56—As2—F57 | 178.10(19) | F86—As7—F88 | 90.22(15) |
| F19—Pt1—F18 | 89.82(12) | F56—As2—F58 | 91.0(2) | F86—As7—F89 | 178.60(17) |
| F19—Pt1—F20 | 90.95(13) | F56—As2—F59 | 88.36(19) | F86—As7—F90 | 89.50(14) |
| F19—Pt1—F21 | 91.26(15) | F56—As2—F60 | 89.52(19) | F87—As7—F88 | 90.02(15) |
| F20—Pt1—F14 | 90.13(13) | F57—As2—F58 | 90.4(2) | F87—As7—F89 | 90.70(16) |
| F20—Pt1—F16 | 90.79(13) | F57—As2—F59 | 90.30(17) | F87—As7—F90 | 179.40(15) |
| F20—Pt1—F18 | 178.59(13) | F57—As2—F60 | 89.13(19) | F88—As7—F89 | 88.65(15) |
| F20—Pt1—F21 | 91.87(13) | F58—As2—F59 | 179.06(19) | F88—As7—F90 | 89.50(14) |
| F21—Pt1—F14 | 177.80(13) | F58—As2—F60 | 89.62(17) | F89—As7—F90 | 89.66(15) |
| F21—Pt1—F16 | 89.00(14) | F59—As2—F60 | 89.72(16) | F91—As8—F92 | 178.34(16) |
| F21—Pt1—F18 | 89.29(12) | F61—As3—F62 | 91.17(18) | F91—As8—F93 | 91.31(16) |
| F23—Pt2—F25 | 88.10(11) | F61—As3—F63 | 91.23(18) | F91—As8—F94 | 89.29(16) |
| F23—Pt2—F27 | 89.32(12) | F61—As3—F64 | 178.48(17) | F91—As8—F95 | 89.34(18) |
| F25—Pt2—F27 | 88.93(11) | F61—As3—F65 | 90.15(17) | F91—As8—F96 | 90.75(16) |
| F28—Pt2—F23 | 90.74(13) | F61—As3—F66 | 90.01(17) | F92—As8—F94 | 89.07(16) |
| F28—Pt2—F25 | 90.08(12) | F62—As3—F63 | 90.13(17) | F92—As8—F95 | 90.46(18) |
| F28—Pt2—F27 | 179.00(12) | F62—As3—F64 | 90.04(16) | F92—As8—F96 | 89.47(16) |
| F28—Pt2—F29 | 91.23(13) | F62—As3—F65 | 178.61(18) | F93—As8—F92 | 90.34(17) |
| F28—Pt2—F30 | 91.28(13) | F62—As3—F66 | 90.09(16) | F93—As8—F94 | 177.69(17) |
| F29—Pt2—F23 | 91.11(12) | F63—As3—F64 | 89.68(16) | F93—As8—F95 | 90.61(19) |
| F29—Pt2—F25 | 178.48(11) | F63—As3—F65 | 90.29(18) | F93—As8—F96 | 88.70(15) |
| F29—Pt2—F27 | 89.76(12) | F63—As3—F66 | 178.74(16) | F94—As8—F95 | 91.6(2) |
| F29—Pt2—F30 | 90.82(12) | F64—As3—F65 | 88.63(16) | F94—As8—F96 | 89.05(16) |
| F30—Pt2—F23 | 177.18(12) | F64—As3—F66 | 89.08(15) | F95—As8—F96 | 179.31(19) |
| F30—Pt2—F25 | 89.92(11) | F65—As3—F66 | 89.46(17) | Xe9—F14—Pt1—F20 | 3.9(3) |
| F30—Pt2—F27 | 88.63(12) | F67—As4—F68 | 90.51(17) | Xe10—F16—Pt1—F21 | −26.8(3) |
| F32—Pt3—F34 | 91.63(11) | F67—As4—F69 | 90.30(17) | Xe11—F18—Pt1—F19 | 1.91(19) |
| F32—Pt3—F36 | 87.62(11) | F67—As4—F70 | 91.27(19) | Xe12—F23—Pt2—F28 | 21.9(2) |
| F36—Pt3—F34 | 89.13(10) | F67—As4—F71 | 177.90(17) | Xe13—F25—Pt2—F30 | 25.50(16) |
| F37—Pt3—F32 | 90.48(11) | F67—As4—F72 | 89.45(16) | Xe14—F27—Pt2—F29 | −2.23(18) |
| F37—Pt3—F34 | 90.06(11) | F68—As4—F69 | 178.98(18) | Xe15—F32—Pt3—F37 | 4.76(18) |
| F37—Pt3—F36 | 177.92(11) | F68—As4—F70 | 90.26(17) | Xe16—F34—Pt3—F38 | −18.85(17) |
| F37—Pt3—F38 | 90.03(12) | F68—As4—F71 | 89.74(16) | Xe17—F36—Pt3—F39 | −4.32(17) |
| F37—Pt3—F39 | 91.44(12) | F68—As4—F72 | 89.76(16) | Xe18—F41—Pt4—F48 | 41.5(2) |
| F38—Pt3—F32 | 178.35(12) | F69—As4—F70 | 90.33(17) | Xe19—F43—Pt4—F47 | −28.9(2) |
| F38—Pt3—F34 | 86.80(11) | F69—As4—F71 | 89.42(17) | Xe20—F45—Pt4—F47 | 26.1(2) |

**Table S4.** Experimental (SCXRD) geometrical parameters for $[Xe_2F_3][PdF_3(XeF_2)_3][AsF_6]_2$.

| Bond distances (Å) | | | | | |
|---|---|---|---|---|---|
| Xe1—F1 | 1.915(4) | Pd1—F5 | 1.990(4) | As1—F16 | 1.726(3) |
| Xe1—F2 | 2.142(4) | Pd1—F7 | 1.975(4) | As1—F17 | 1.731(4) |
| Xe2—F2 | 2.162(4) | Pd1—F9 | 1.979(4) | As1—F18 | 1.737(3) |
| Xe2—F3 | 1.916(4) | Pd1—F10 | 1.842(5) | As2—F19 | 1.714(4) |
| Xe3—F4 | 1.918(4) | Pd1—F11 | 1.856(4) | As2—F20 | 1.717(4) |
| Xe3—F5 | 2.143(4) | Pd1—F12 | 1.860(4) | As2—F21 | 1.724(4) |
| Xe4—F6 | 1.920(4) | As1—F13 | 1.717(4) | As2—F22 | 1.724(4) |
| Xe4—F7 | 2.178(4) | As1—F14 | 1.721(5) | As2—F23 | 1.728(4) |
| Xe5—F8 | 1.923(4) | As1—F15 | 1.726(3) | As2—F24 | 1.740(4) |
| Xe5—F9 | 2.155(4) | | | | |
| **Angles (°)** | | | | | |
| F1—Xe1—F2 | 179.36(19) | F11—Pd1—F9 | 90.6(2) | F17—As1—F18 | 89.4(3) |
| F3—Xe2—F2 | 178.1(2) | F11—Pd1—F12 | 90.5(2) | F19—As2—F20 | 90.2(3) |
| Xe1—F2—Xe2 | 133.11(19) | F12—Pd1—F5 | 88.2(2) | F19—As2—F21 | 91.1(3) |
| F4—Xe3—F5 | 177.2(2) | F12—Pd1—F7 | 179.5(2) | F19—As2—F22 | 91.2(3) |
| F6—Xe4—F7 | 178.2(2) | F12—Pd1—F9 | 88.3(2) | F19—As2—F23 | 178.6(3) |
| F8—Xe5—F9 | 177.80(18) | F13—As1—F14 | 90.4(4) | F19—As2—F24 | 90.1(2) |
| Pd1—F5—Xe3 | 130.6(2) | F13—As1—F15 | 90.6(3) | F20—As2—F21 | 90.6(2) |
| Pd1—F7—Xe4 | 125.31(18) | F13—As1—F16 | 90.8(3) | F20—As2—F22 | 90.7(3) |
| Pd1—F9—Xe5 | 124.3(2) | F13—As1—F17 | 90.6(3) | F20—As2—F23 | 90.4(2) |
| F7—Pd1—F5 | 91.69(17) | F13—As1—F18 | 179.7(3) | F20—As2—F24 | 179.6(2) |
| F7—Pd1—F9 | 91.21(18) | F14—As1—F15 | 90.5(2) | F21—As2—F22 | 177.4(2) |
| F9—Pd1—F5 | 88.02(18) | F14—As1—F16 | 90.0(2) | F21—As2—F23 | 90.3(2) |
| F10—Pd1—F5 | 90.8(2) | F14—As1—F17 | 178.9(3) | F21—As2—F24 | 89.1(2) |
| F10—Pd1—F7 | 87.9(2) | F14—As1—F18 | 89.6(3) | F22—As2—F23 | 87.5(2) |
| F10—Pd1—F9 | 178.4(2) | F15—As1—F16 | 178.5(2) | F22—As2—F24 | 89.6(2) |
| F10—Pd1—F11 | 90.6(3) | F15—As1—F17 | 90.0(2) | F23—As2—F24 | 89.4(2) |
| F10—Pd1—F12 | 92.6(3) | F15—As1—F18 | 89.0(2) | Xe3—F5—Pd1—F10 | −42.6(4) |
| F11—Pd1—F5 | 178.16(19) | F16—As1—F17 | 89.5(2) | Xe4—F7—Pd1—F11 | 13.9(3) |
| F11—Pd1—F7 | 89.61(19) | F16—As1—F18 | 89.5(2) | Xe5—F9—Pd1—F12 | 47.3(3) |

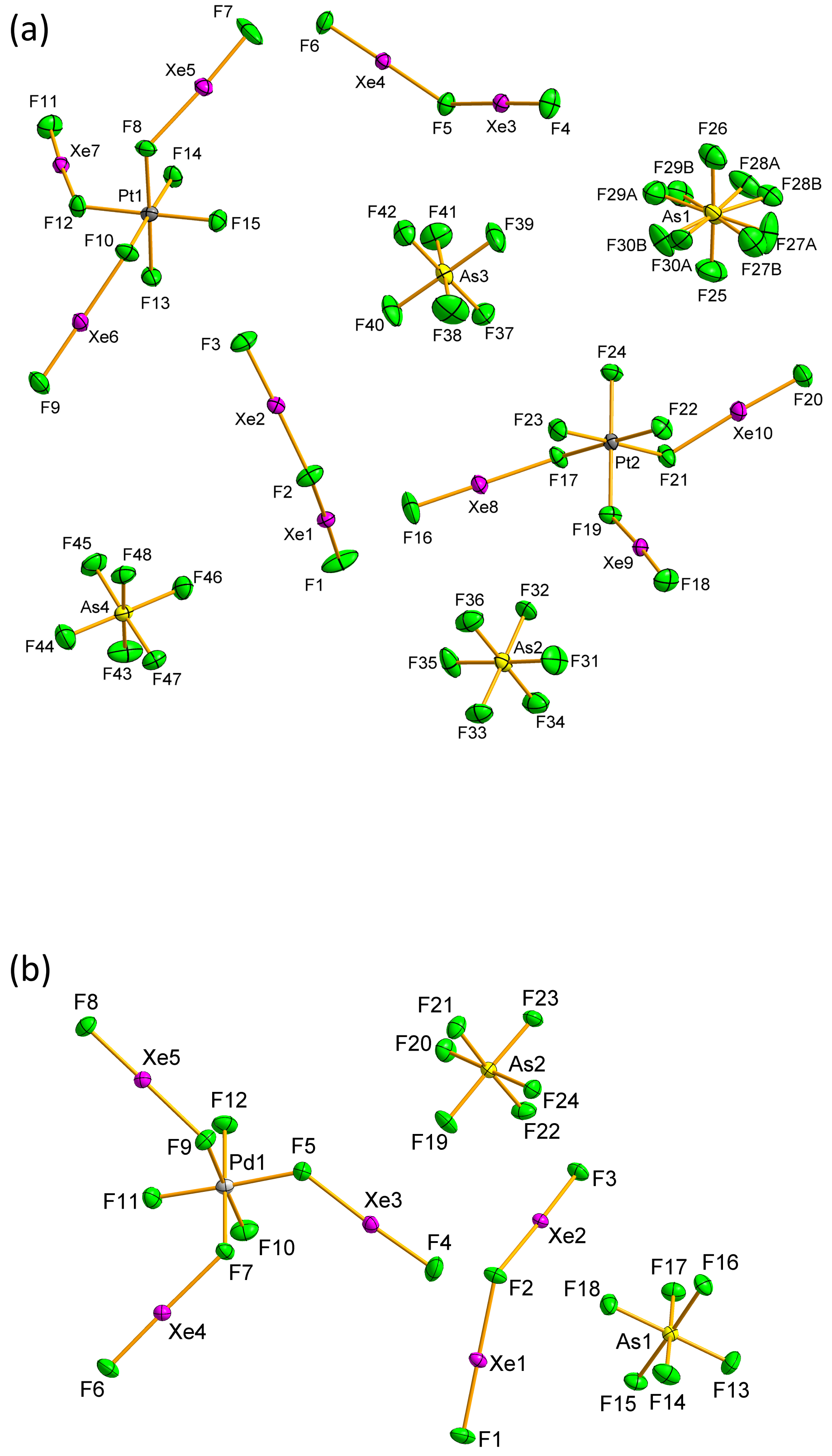


**Figure S2.** (a) Composite view of the asymmetric unit (*Z'* = 2) of $[Xe_2F_3][PtF_3(XeF_2)_3][AsF_6]_2$(*oP*256); (b) view of the asymmetric unit (*Z'* = 1) of $[Xe_2F_3][PdF_3(XeF_2)_3][AsF_6]_2$, showing atom labeling. Displacement ellipsoids are drawn at the 50% probability level. Fluorine atoms of the $[As1F_6]^-$ anion in $[Xe_2F_3][PtF_3(XeF_2)_3][AsF_6]_2$(*oP*256) are disordered over two positions (0.56(2):0.44(2)).

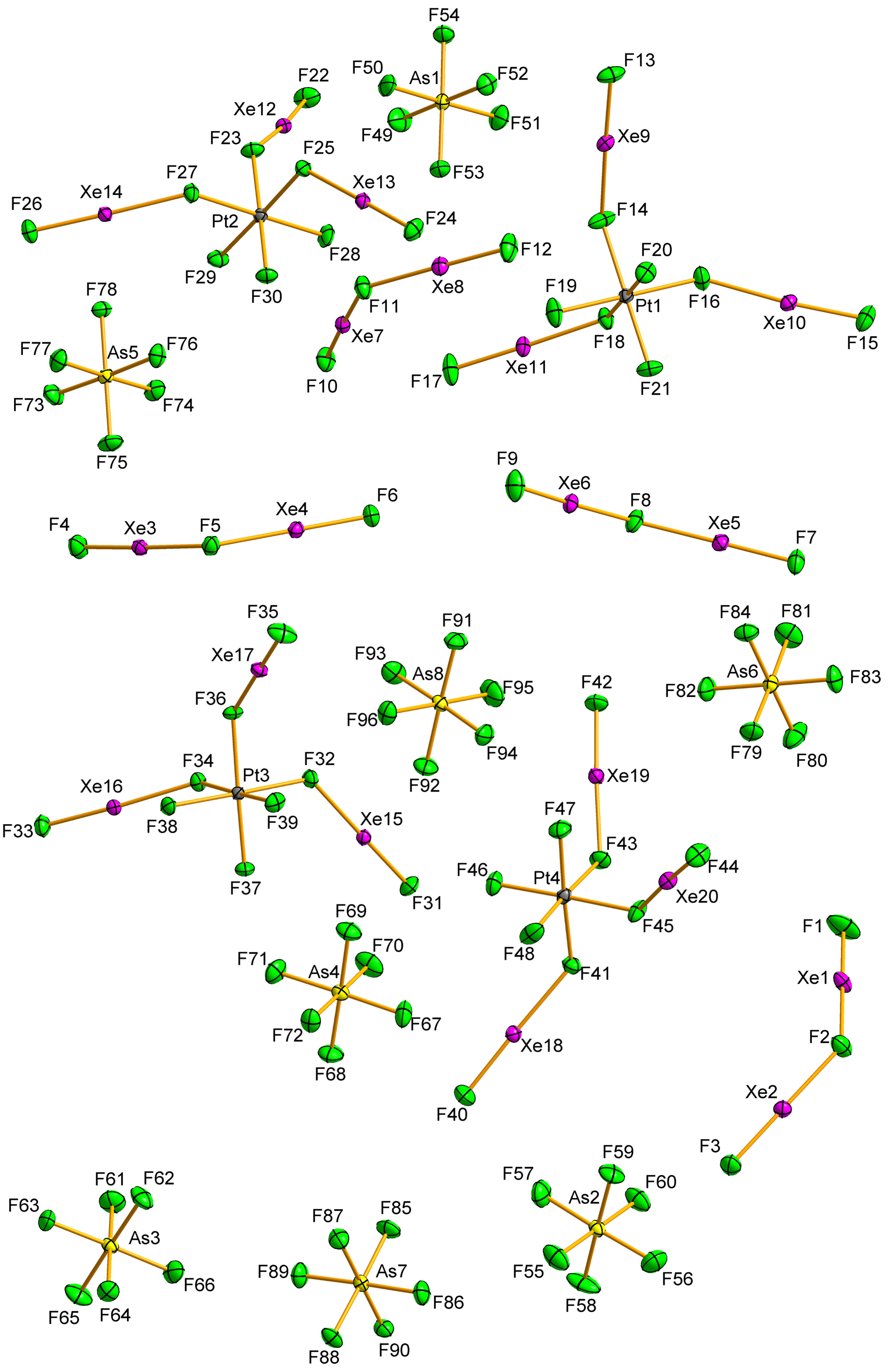


**Figure S3.** Composite view of the asymmetric unit ($Z'$ = 4) of $[Xe_2F_3][PtF_3(XeF_2)_3][AsF_6]_2$($aP$256), showing atom labeling. Displacement ellipsoids are drawn at the 50% probability level.

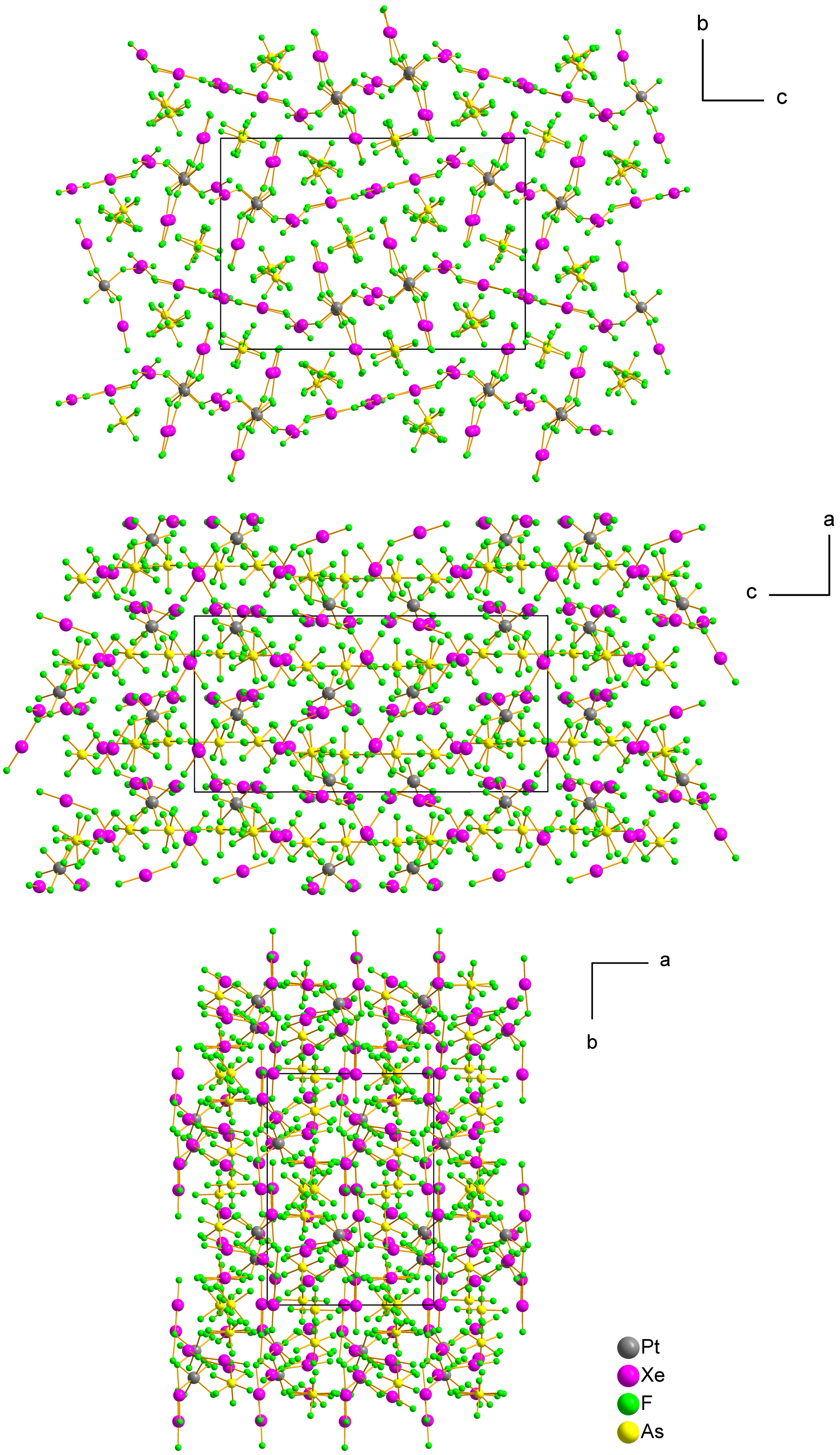


**Figure S4.** The crystal packing and the unit cell of $[Xe_2F_3][PtF_3(XeF_2)_3][AsF_6]_2$(*oP*256) viewed along the *a*- (top), *b*- (middle), and *c*-crystallographic axis (bottom).

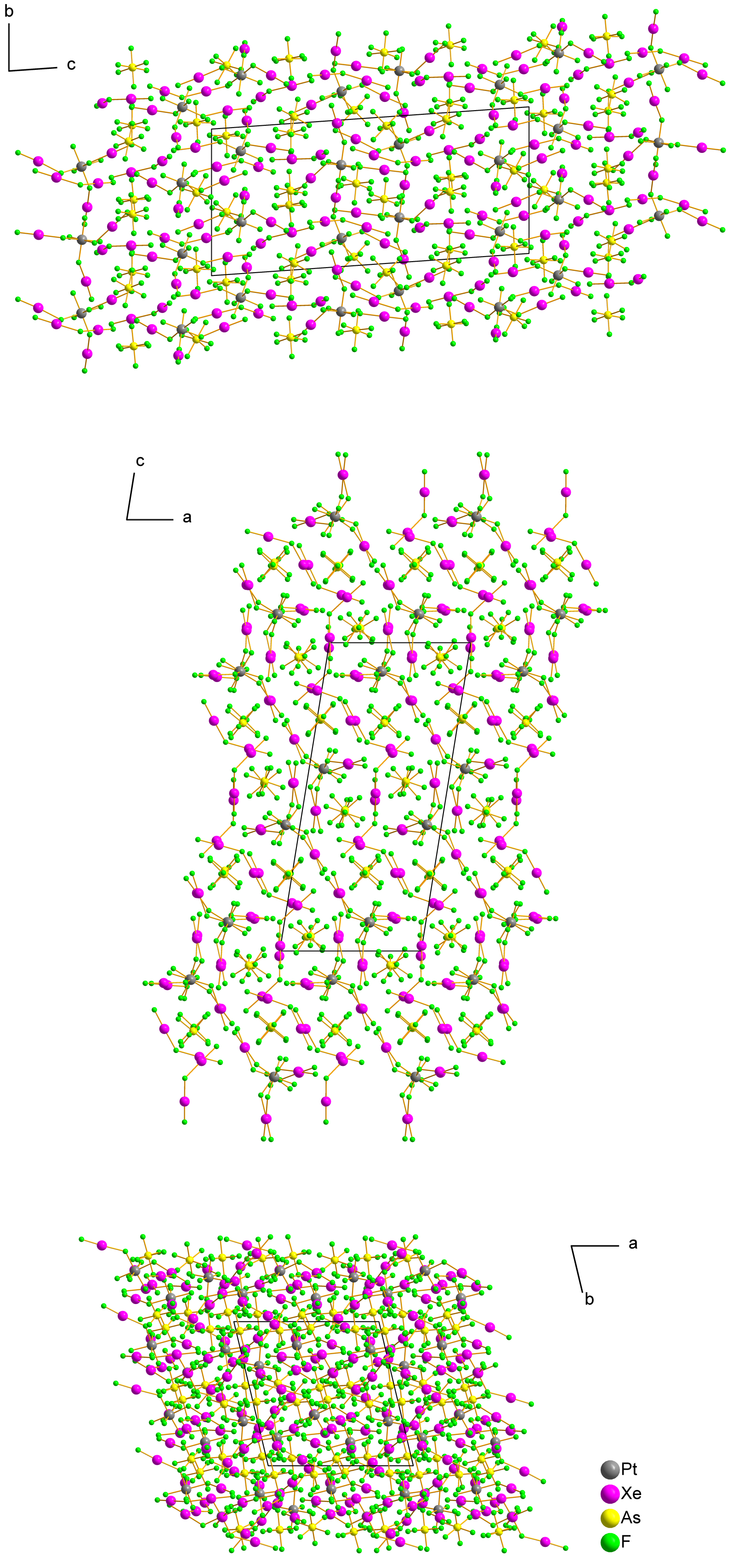


**Figure S5.** The crystal packing and the unit cell of $[Xe_2F_3][PtF_3(XeF_2)_3][AsF_6]_2$(*aP*256) viewed along the *a*- (top), *b*- (middle), and *c*-crystallographic axis (bottom).

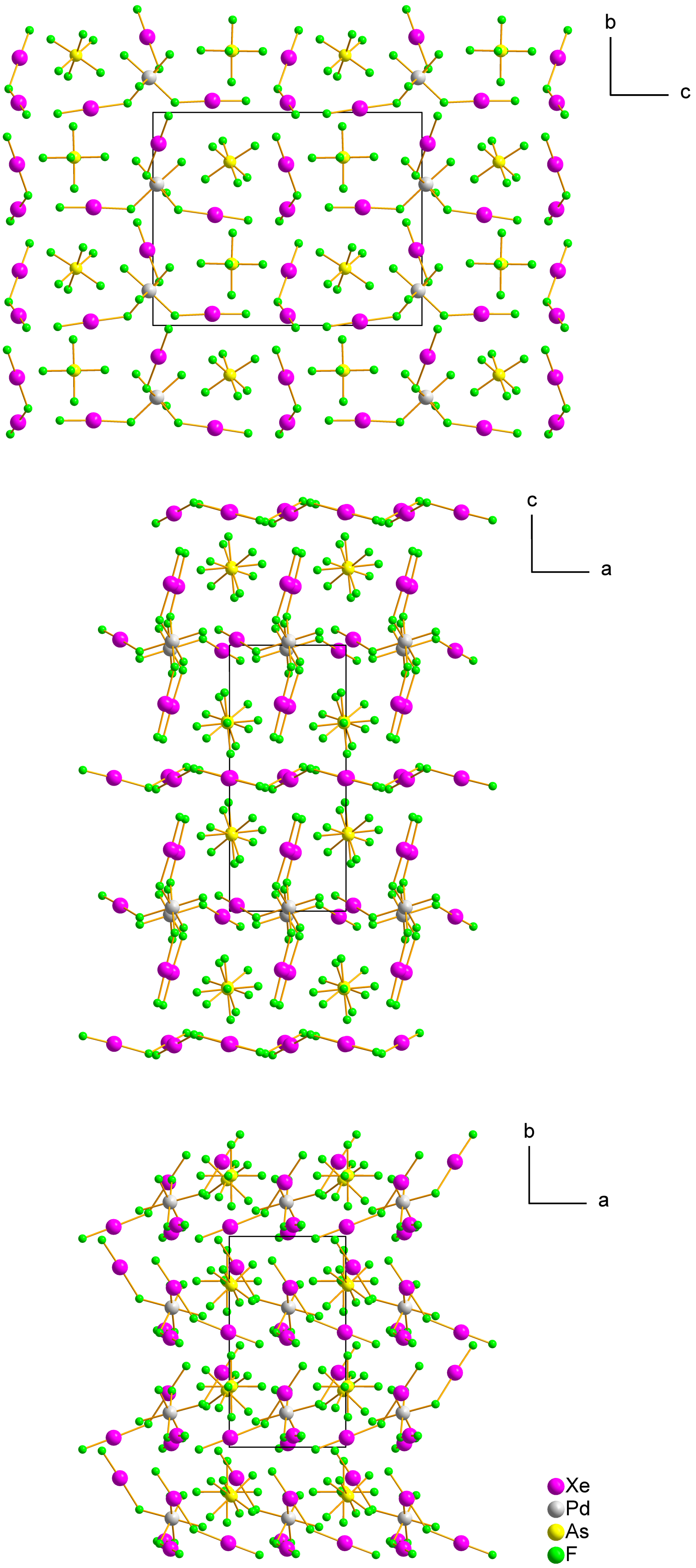


**Figure S6.** The crystal packing and the unit cell of $[Xe_2F_3][PdF_3(XeF_2)_3][AsF_6]_2$ viewed along the *a*- (top), *b*- (middle), and *c*-crystallographic axis (bottom).

**Table S5.** Non-bonded inter- and intramolecular Xe···F contacts shorter than the sum of van der Waals radii for Xe and F (3.74 Å)[1,2] in the crystal structure of $[Xe_2F_3][PtF_3(XeF_2)_3][AsF_6]_2$(*oP*256). Due to the substantial number of symmetry-independent contacts, only the total number of contacts as well as the shortest and longest contact for each Xe atom are tabulated.

| Atom | Nr. of contacts | Distance to closest (Å) | Closest F atom | Distance to furthest (Å) | Furthest F atom |
|---|---|---|---|---|---|
| Xe1 | 11 | 3.085(17) | F30B | 3.604(8) | $F16^{i}$ |
| Xe2 | 11 | 3.122(5) | $F48^{ii}$ | 3.624(6) | $F45^{ii}$ |
| Xe3 | 9 | 3.048(5) | $F43^{iii}$ | 3.603(7) | F35 |
| Xe4 | 12 | 3.065(8) | $F39^{iv}$ | 3.563(8) | $F25^{v}$ |
| Xe5 | 11 | 3.291(7) | F42 | 3.73(2) | F15 |
| Xe6 | 8 | 3.135(5) | $F47^{vi}$ | 3.590(7) | $F11^{vii}$ |
| Xe7 | 7 | 3.035(7) | F31 | 3.364(5) | $F24^{viii}$ |
| Xe8 | 9 | 3.048(14) | $F29A^{viii}$ | 3.689(7) | $F4^{ix}$ |
| Xe9 | 9 | 3.096(15) | $F28B^{viii}$ | 3.708(5) | $F48^{iii}$ |
| Xe10 | 10 | 2.945(18) | $F28A^{viii}$ | 3.647(6) | $F46^{iii}$ |

Symmetry codes: (i) *x*, *y* + 1, *z*; (ii) *x* – 1/2, –*y* + 3/2, –*z* + 1; (iii) –*x* + 3/2, –*y* + 1, *z* – 1/2; (iv) *x* + 1, *y*, *z*; (v) *x* + 1/2, –*y* + 3/2, –*z* + 1; (vi) –*x* + 1, *y* – 1/2, –*z* + 3/2; (vii) *x* – 1/2, –*y* + 1/2, –*z* + 1; (viii) *x* + 1/2, –*y* + 1/2, –*z* + 1; (ix) –*x* + 1, *y* – 1/2, –*z* + 1/2.

**Table S6.** Non-bonded inter- and intramolecular Xe···F contacts shorter than the sum of van der Waals radii for Xe and F (3.74 Å)[1,2] in the crystal structure of $[Xe_2F_3][PtF_3(XeF_2)_3][AsF_6]_2$(*aP*256). Due to the substantial number of symmetry-independent contacts, only the total number of contacts as well as the shortest and longest contact for each Xe atom are tabulated.

| Atom | Nr. of contacts | Distance to closest (Å) | Closest F atom | Distance to furthest (Å) | Furthest F atom |
|---|---|---|---|---|---|
| Xe1 | 8 | 3.068(3) | F54$^{i}$ | 3.728(4) | F52$^{i}$ |
| Xe2 | 10 | 3.201(4) | F96 | 3.718(4) | F89 |
| Xe3 | 9 | 3.113(3) | F78 | 3.537(3) | F88$^{ii}$ |
| Xe4 | 9 | 3.171(4) | F63$^{iii}$ | 3.664(4) | F61$^{ii}$ |
| Xe5 | 9 | 3.175(4) | F56 | 3.672(4) | F58 |
| Xe6 | 10 | 3.123(3) | F53$^{iv}$ | 3.720(4) | F51$^{iv}$ |
| Xe7 | 11 | 3.075(3) | F90 | 3.700(4) | F89 |
| Xe8 | 9 | 3.157(3) | F90 | 3.724(4) | F49 |
| Xe9 | 9 | 3.087(3) | F83$^{v}$ | 3.657(3) | F79$^{vi}$ |
| Xe10 | 10 | 3.075(4) | F48$^{vii}$ | 3.641(3) | F18$^{v}$ |
| Xe11 | 9 | 3.121(4) | F72$^{iii}$ | 3.614(4) | F9 |
| Xe12 | 9 | 3.118(4) | F49 | 3.668(4) | F28 |
| Xe13 | 10 | 3.062(4) | F93 | 3.711(4) | F17$^{vi}$ |
| Xe14 | 11 | 2.983(3) | F78 | 3.727(3) | F77 |
| Xe15 | 10 | 3.110(3) | F92 | 3.718(3) | F3 |
| Xe16 | 11 | 3.027(3) | F38$^{viiii}$ | 3.590(3) | F77 |
| Xe17 | 9 | 3.118(3) | F33$^{viiii}$ | 3.656(4) | F62$^{vi}$ |
| Xe18 | 10 | 3.084(4) | F67 | 3.715(4) | F46 |
| Xe19 | 8 | 3.164(3) | F85 | 3.645(4) | F47 |
| Xe20 | 7 | 3.029(3) | F84$^{v}$ | 3.705(3) | F41$^{vii}$ |

Symmetry codes: (i) *x*, *y* – 1, *z*; (ii) –*x* + 1, –*y* + 1, –*z* + 1; (iii) *x*, *y* + 1, *z*; (iv) *x* – 1, *y*, *z*; (v) –*x* + 1, –*y* + 2, –*z*; (vi) *x* + 1, *y*, *z*; (vii) –*x* + 1, –*y* + 1, –*z*; (viii) –*x* + 2, –*y* + 1, –*z* + 1.

**Table S7.** Non-bonded inter- and intramolecular Xe···F contacts shorter than the sum of van der Waals radii for Xe and F (3.74 Å)[1,2] in the crystal structure of $[Xe_2F_3][PdF_3(XeF_2)_3][AsF_6]_2$. Due to the substantial number of symmetry-independent contacts, only the total number of contacts as well as the shortest and longest contact for each Xe atom are tabulated.

| Atom | Nr. of contacts | Distance to closest (Å) | Closest F atom | Distance to furthest (Å) | Furthest F atom |
|---|---|---|---|---|---|
| Xe1 | 10 | 3.122(4) | F24$^{i}$ | 3.677(6) | F18$^{ii}$ |
| Xe2 | 10 | 3.025(5) | F22 | 3.548(5) | F8$^{iii}$ |
| Xe3 | 8 | 3.184(5)$^{iv}$ | F20 | 3.725(4) | F7 |
| Xe4 | 9 | 3.139(5) | F13$^{ii}$ | 3.683(6) | F19$^{v}$ |
| Xe5 | 9 | 3.107(4) | F23$^{vi}$ | 3.672(5) | F15$^{vii}$ |

Symmetry codes: (i) –*x* + 1, *y* + 1/2, –*z* + 1; (ii) –*x*, *y* + 1/2, –*z* + 1; (iii) *x*, *y*, *z* – 1; (iv) *x* – 1, *y*, *z*; (v) –*x* + 1, *y* + 1/2, –*z* + 2; (vi) *x* – 2, *y* + 1/2, –*z* + 2; (vii) *x* + 1, *y*, *z* + 1.

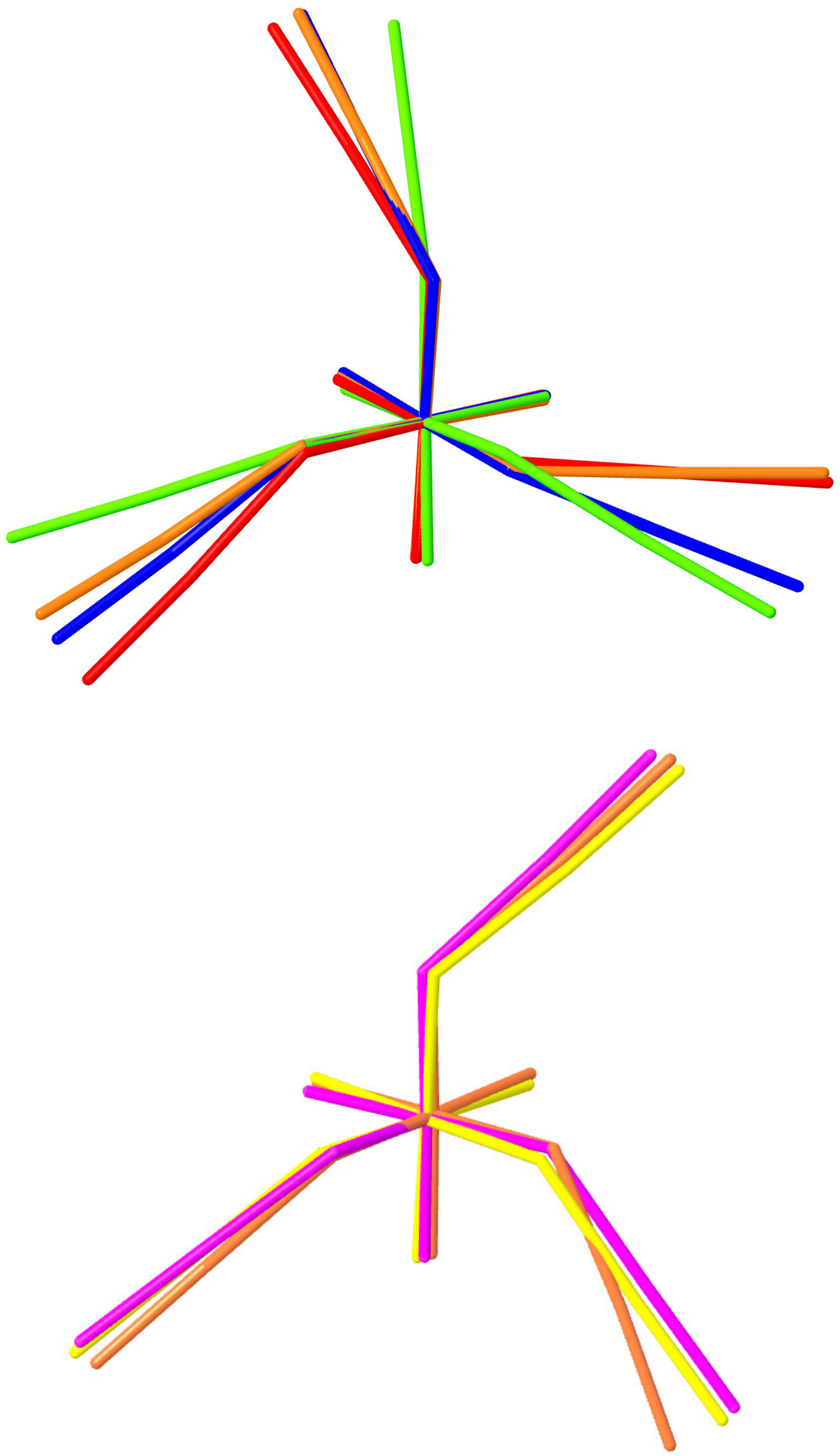

**Figure S7.** Overlay of crystallographically independent $[PtF_3(XeF_2)_3]^+$ adduct cations found in the crystal structure of $[Xe_2F_3][PtF_3(XeF_2)_3][AsF_6]_2$(*aP*256) (top); and overlay of the $[PtF_3(XeF_2)_3]^+$ (yellow, orange) and $[PdF_3(XeF_2)_3]^+$ (pink) cations from the $[Xe_2F_3][PtF_3(XeF_2)_3][AsF_6]_2$(*oP*256) and $[Xe_2F_3][PdF_3(XeF_2)_3][AsF_6]_2$ crystal structures, respectively, showing the similarities in their conformations (bottom).

**Table S8.** Calculated (PBE0-D3/aug-cc-pVQZ(-PP)) vibrational frequencies of the $[MF_3(XeF_2)_3]^+$ cations.

| $[PtF_3(XeF_2)_3]^+$ | | | | $[PdF_3(XeF_2)_3]^+$ | | |
|---|---|---|---|---|---|---|
| Frequency ($cm^{-1}$) | IR Intensity [a] | Raman Activity [b] | Vibrational Mode [c] | Frequency ($cm^{-1}$) | IR Intensity [a] | Raman Activity [b] |
| **7** | 0.15 | 1.57 | 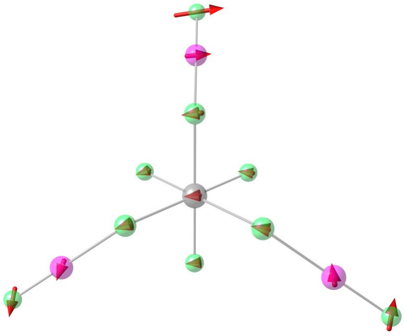 | **13** | 0.10 | 1.67 |
| **16** | 0.20 | 1.66 | 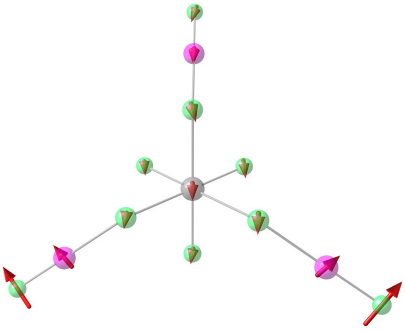 | **18** | 0.20 | 1.52 |
| **30** | 1.05 | 0.85 | 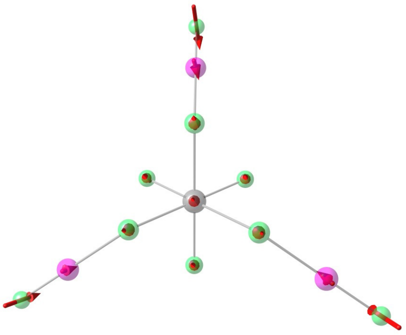 | **32** | 0.54 | 0.56 |
| **51** | 0.27 | 0.02 | 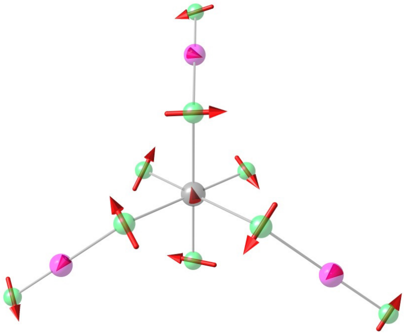 | **40** | 0.20 | 0.09 |
| **61** | 4.93 | 0.45 | 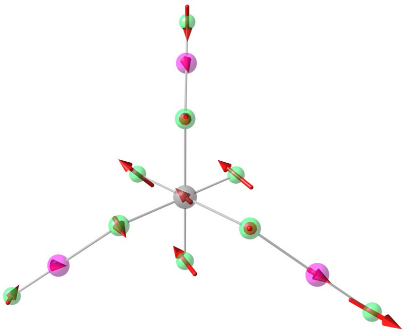 | **59** | 4.59 | 0.24 |

[a] IR intensities in km/mol. [b] Raman activities in $Å^4$/amu. [c] Vibration modes for Pd are analogous.

Table S8. Calculated vibrational frequencies of the $[MF_3(XeF_2)_3]^+$ cations. *(continued)*

| $[PtF_3(XeF_2)_3]^+$ | | | | $[PdF_3(XeF_2)_3]^+$ | | |
|---|---|---|---|---|---|---|
| Frequency ($cm^{-1}$) | IR Intensity [a] | Raman Activity [b] | Vibrational Mode [c] | Frequency ($cm^{-1}$) | IR Intensity [a] | Raman Activity [b] |
| **63** | 4.63 | 0.47 | 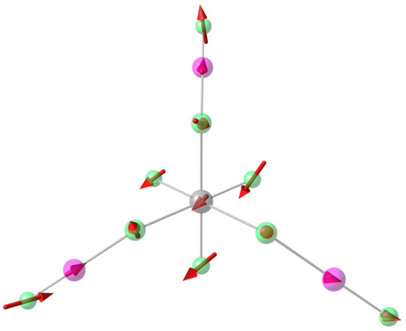 | **62** | 4.10 | 0.26 |
| **82** | 3.12 | 2.85 | 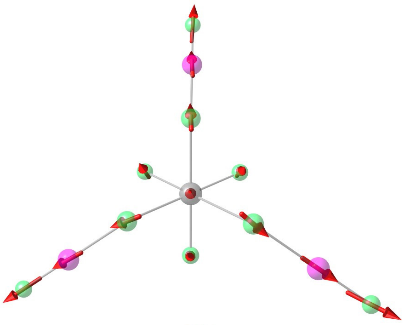 | **86** | 2.29 | 2.15 |
| **103** | 1.22 | 0.23 | 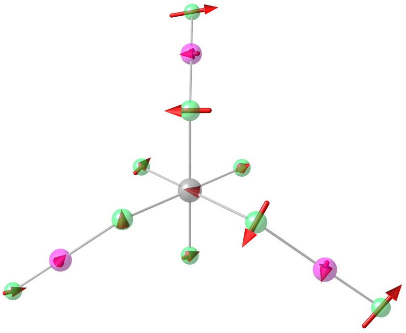 | **107** | 0.15 | 0.13 |
| **106** | 0.59 | 0.24 | 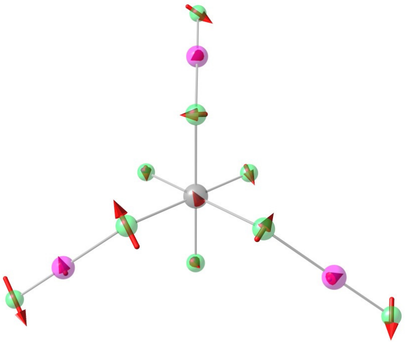 | **107** | 0.40 | 0.24 |
| **139** | 3.35 | 1.91 | 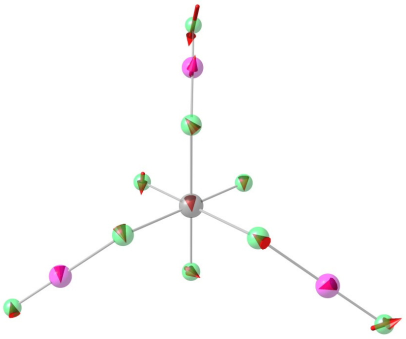 | **142** | 1.61 | 2.75 |

[a] IR intensities in km/mol. [b] Raman activities in $Å^4$/amu. [c] Vibration modes for Pd are analogous.

Table S8. Calculated vibrational frequencies of the $[MF_3(XeF_2)_3]^+$ cations. *(continued)*

| $[PtF_3(XeF_2)_3]^+$ | | | | $[PdF_3(XeF_2)_3]^+$ | | |
|---|---|---|---|---|---|---|
| Frequency ($cm^{-1}$) | IR Intensity [a] | Raman Activity [b] | Vibrational Mode [c] | Frequency ($cm^{-1}$) | IR Intensity [a] | Raman Activity [b] |
| **143** | 3.68 | 2.08 | 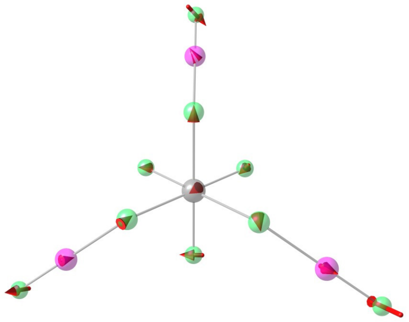 | **143** | 1.58 | 2.79 |
| **144** | 7.03 | 1.59 | 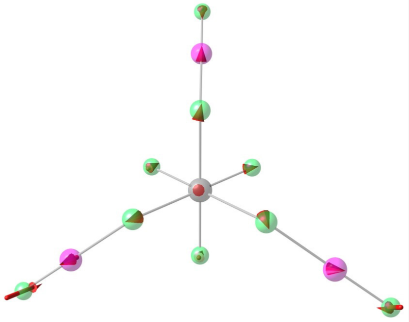 | **146** | 6.93 | 1.70 |
| **147** | 0.49 | 0.31 | 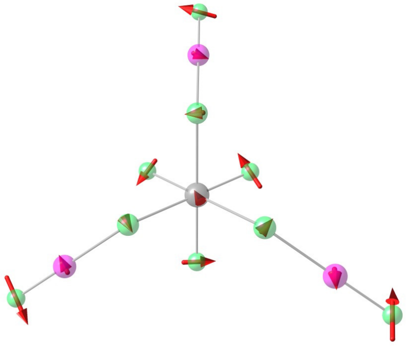 | **148** | 1.89 | 0.47 |
| **177** | 15.01 | 0.43 | 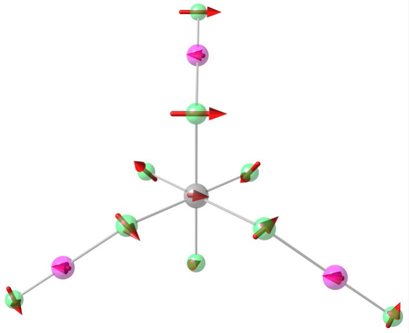 | **182** | 16.50 | 0.23 |
| **178** | 15.06 | 0.40 | 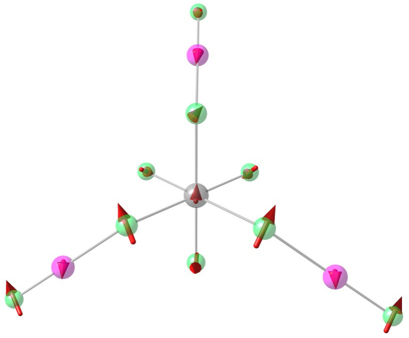 | **183** | 15.15 | 0.12 |

[a] IR intensities in km/mol. [b] Raman activities in Å$^4$/amu. [c] Vibration modes for Pd are analogous.

Table S8. Calculated vibrational frequencies of the $[MF_3(XeF_2)_3]^+$ cations. *(continued)*

| $[PtF_3(XeF_2)_3]^+$ | | | | $[PdF_3(XeF_2)_3]^+$ | | |
|---|---|---|---|---|---|---|
| Frequency ($cm^{-1}$) | IR Intensity [a] | Raman Activity [b] | Vibrational Mode [c] | Frequency ($cm^{-1}$) | IR Intensity [a] | Raman Activity [b] |
| **218** | 0.59 | 3.30 | 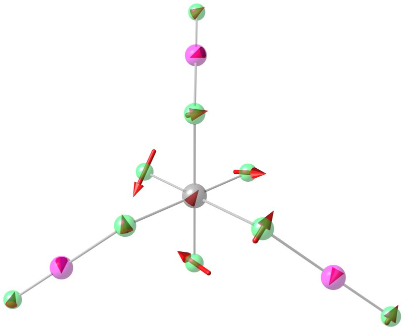 | **230** | 0.20 | 4.14 |
| **218** | 0.57 | 3.31 | 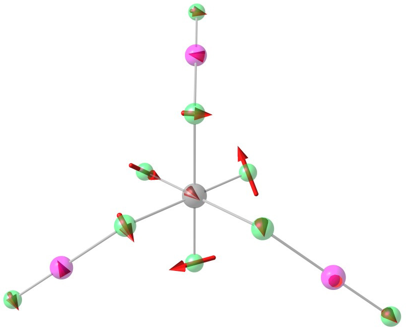 | **232** | 0.40 | 3.44 |
| **228** | 0.20 | 2.03 | 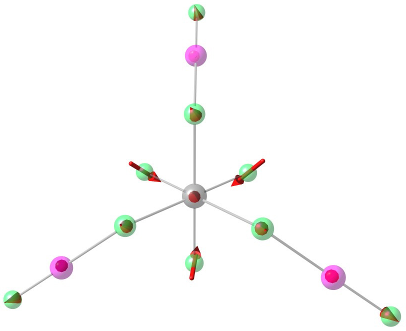 | **245** | 0.19 | 0.08 |
| **254** | 0.01 | 0.01 | 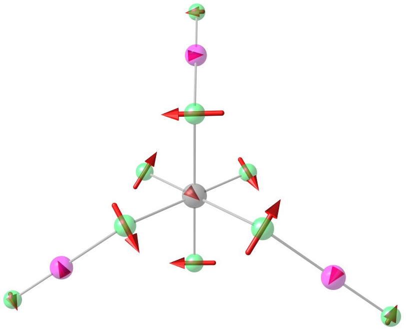 | **249** | 0.02 | 3.77 |
| **260** | 0.29 | 1.23 | 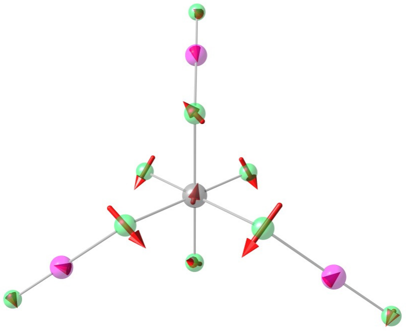 | **277** | 0.32 | 2.08 |

[a] IR intensities in km/mol. [b] Raman activities in Å$^4$/amu. [c] Vibration modes for Pd are analogous.

Table S8. Calculated vibrational frequencies of the $[MF_3(XeF_2)_3]^+$ cations. *(continued)*

| $[PtF_3(XeF_2)_3]^+$ | | | | $[PdF_3(XeF_2)_3]^+$ | | |
|---|---|---|---|---|---|---|
| Frequency ($cm^{-1}$) | IR Intensity [a] | Raman Activity [b] | Vibrational Mode [c] | Frequency ($cm^{-1}$) | IR Intensity [a] | Raman Activity [b] |
| **263** | 0.14 | 1.36 | 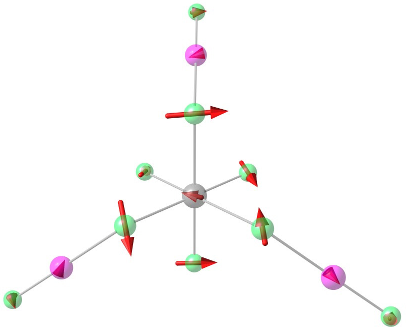 | **280** | 0.43 | 2.38 |
| **372** | 74.71 | 7.37 | 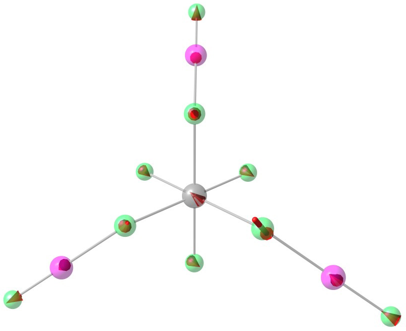 | **368** | 77.82 | 2.67 |
| **373** | 70.87 | 7.19 | 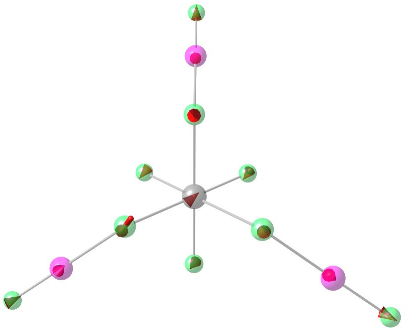 | **370** | 71.37 | 2.12 |
| **390** | 23.37 | 44.02 | 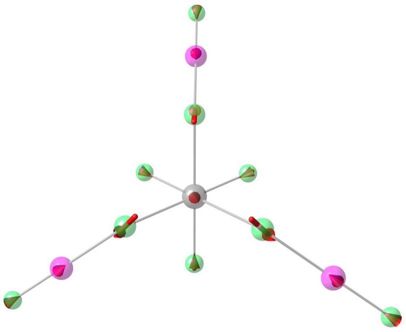 | **398** | 19.54 | 26.84 |
| **498** | 333.56 | 7.15 | 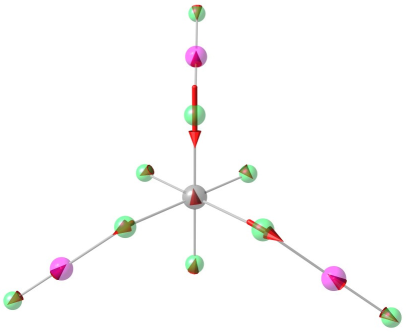 | **478** | 384.78 | 0.01 |

[a] IR intensities in km/mol. [b] Raman activities in $Å^4$/amu. [c] Vibration modes for Pd are analogous.

Table S8. Calculated vibrational frequencies of the $[MF_3(XeF_2)_3]^+$ cations. *(continued)*

| $[PtF_3(XeF_2)_3]^+$ | | | | $[PdF_3(XeF_2)_3]^+$ | | |
|---|---|---|---|---|---|---|
| Frequency ($cm^{-1}$) | IR Intensity [a] | Raman Activity [b] | Vibrational Mode [c] | Frequency ($cm^{-1}$) | IR Intensity [a] | Raman Activity [b] |
| **499** | 331.12 | 7.33 | 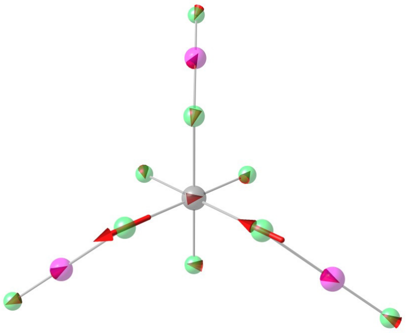 | 483 | 376.54 | 0.06 |
| **508** | 24.10 | 25.32 | 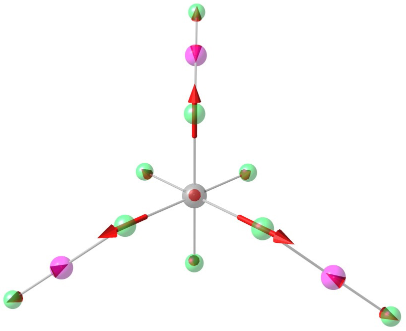 | 503 | 24.60 | 1.85 |
| **624** | 175.97 | 66.94 | 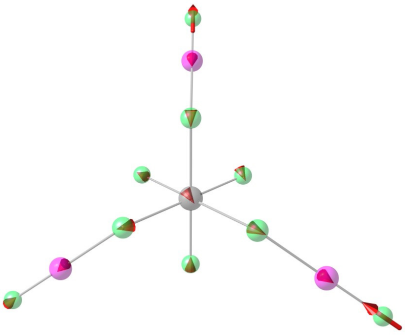 | 624 | 181.43 | 44.17 |
| **625** | 165.61 | 70.41 | 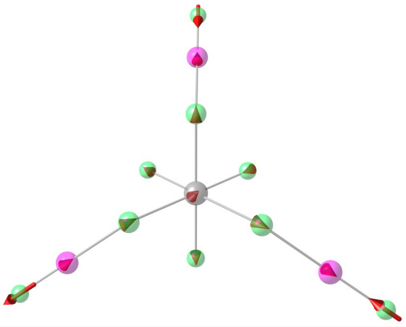 | 625 | 176.53 | 45.56 |
| **629** | 23.12 | 121.44 | 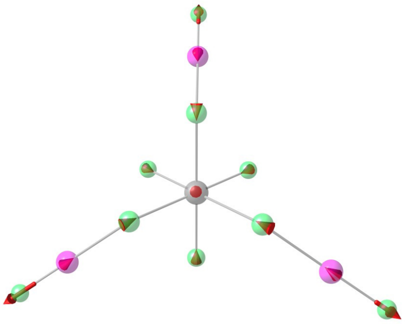 | 629 | 12.87 | 74.70 |

[a] IR intensities in km/mol. [b] Raman activities in $Å^4$/amu. [c] Vibration modes for Pd are analogous.

Table S8. Calculated vibrational frequencies of the $[MF_3(XeF_2)_3]^+$ cations. *(continued)*

| $[PtF_3(XeF_2)_3]^+$ | | | | $[PdF_3(XeF_2)_3]^+$ | | |
|---|---|---|---|---|---|---|
| Frequency ($cm^{-1}$) | IR Intensity [a] | Raman Activity [b] | Vibrational Mode [c] | Frequency ($cm^{-1}$) | IR Intensity [a] | Raman Activity [b] |
| **666** | 51.54 | 17.26 | 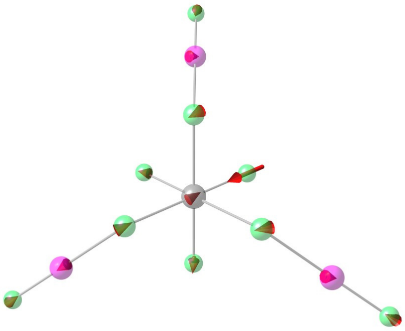 | **666** | 51.47 | 65.75 |
| **667** | 53.29 | 15.94 | 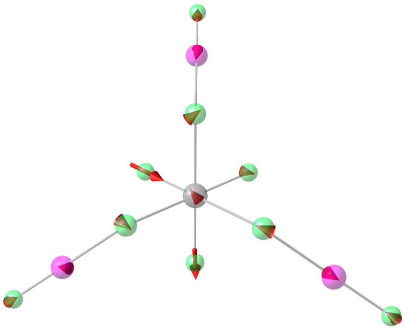 | **670** | 80.64 | 21.44 |
| **669** | 40.95 | 21.92 | 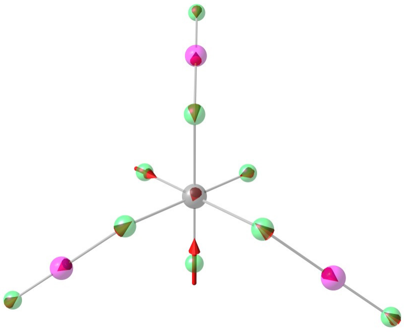 | **671** | 78.06 | 24.96 |

[a] IR intensities in km/mol. [b] Raman activities in $Å^4$/amu. [c] Vibration modes for Pd are analogous.

**Table S9.** Optimized geometry of $[PtF_3(XeF_2)_3]^+$ cation (PBE0-D3/aug-cc-pVQZ(-PP)).

| Atom | *x* | *y* | *z* |
|---|---|---|---|
| Pt | 6.722358 | 2.992413 | 8.492103 |
| Xe | 9.223283 | 1.906559 | 6.113816 |
| Xe | 5.373747 | 0.667506 | 10.906446 |
| Xe | 5.427294 | 6.367708 | 8.362343 |
| F | 11.013416 | 1.625291 | 5.512056 |
| F | 5.348240 | 1.607317 | 8.932607 |
| F | 7.139104 | 2.191851 | 6.707971 |
| F | 5.378517 | 4.271085 | 7.744919 |
| F | 6.320120 | 3.743667 | 10.165850 |
| F | 8.009455 | 4.293510 | 8.070963 |
| F | 7.980608 | 1.783941 | 9.187920 |
| F | 5.408951 | 8.216124 | 8.838506 |
| F | 5.324606 | –0.200373 | 12.607303 |

**Table S10.** Optimized geometry of $[PdF_3(XeF_2)_3]^+$ cation (PBE0-D3/aug-cc-pVQZ(-PP)).

| Atom | *x* | *y* | *z* |
|---|---|---|---|
| Pd | 2.632031 | 7.744586 | 15.164881 |
| Xe | 3.806194 | 6.062465 | 18.121274 |
| Xe | 3.506671 | 6.462009 | 11.917212 |
| Xe | 0.284711 | 10.459114 | 15.406143 |
| F | 2.225468 | 6.504769 | 13.669503 |
| F | 5.023109 | 5.774908 | 19.565134 |
| F | 2.998201 | 8.889689 | 16.564418 |
| F | 2.359666 | 6.324528 | 16.523567 |
| F | 4.578600 | 6.369352 | 10.339294 |
| F | 4.384125 | 7.177657 | 15.049819 |
| F | 0.744978 | 8.339645 | 15.289907 |
| F | 2.870499 | 9.062204 | 13.895514 |
| F | –0.208950 | 12.300672 | 15.508034 |

**Table S11.** Calculated (PBE0-D3/aug-cc-pVQZ(-PP)) geometrical parameters of the $[PtF_3(XeF_2)_3]^+$ cation.

| Bond | Calculated distance (Å) | Mayer bond order | Experimental distance† (Å) | Bond | Calculated distance (Å) | Mayer bond order | Experimental distance† (Å) |
|---|---|---|---|---|---|---|---|
| Xe—$F_t$ | 1.909 | 0.683 | 1.908 | Pt—$F_b$ | 2.000 | 0.579 | 1.989 |
| Xe—$F_b$ | 2.186 | 0.203 | 2.199 | Pt—$F_b$ | 2.000 | 0.579 | 1.989 |
| Xe—$F_t$ | 1.910 | 0.683 | 1.908 | Pt—$F_b$ | 1.999 | 0.579 | 1.989 |
| Xe—$F_b$ | 2.186 | 0.204 | 2.199 | Pt—$F_t$ | 1.878 | 0.918 | 1.889 |
| Xe—$F_t$ | 1.909 | 0.682 | 1.908 | Pt—$F_t$ | 1.878 | 0.921 | 1.889 |
| Xe—$F_b$ | 2.186 | 0.203 | 2.199 | Pt—$F_t$ | 1.878 | 0.920 | 1.889 |

| Angle (°) | Calculated | Experimental† | Angle (°) | Calculated | Experimental† |
|---|---|---|---|---|---|
| $F_t$—Xe—$F_b$ | 177.3 | 176.8 | $F_b$—Pt—$F_t$ | 86.2 | 90.0 |
| $F_t$—Xe—$F_b$ | 177.3 | 176.8 | $F_b$—Pt—$F_t$ | 86.2 | 90.0 |
| $F_t$—Xe—$F_b$ | 177.2 | 176.8 | $F_b$—Pt—$F_t$ | 179.8 | 178.3 |
| Pt—$F_b$—Xe | 119.5 | 128.0 | $F_b$—Pt—$F_t$ | 179.7 | 178.3 |
| Pt—$F_b$—Xe | 119.2 | 128.0 | $F_b$—Pt—$F_t$ | 86.2 | 90.0 |
| Pt—$F_b$—Xe | 119.6 | 128.0 | $F_b$—Pt—$F_t$ | 86.2 | 90.0 |
| $F_b$—Pt—$F_b$ | 93.6 | 90.0 | $F_t$—Pt—$F_t$ | 94.1 | 90.0 |
| $F_b$—Pt—$F_b$ | 93.6 | 90.0 | $F_t$—Pt—$F_t$ | 94.0 | 90.0 |
| $F_b$—Pt—$F_t$ | 86.2 | 90.0 | $F_t$—Pt—$F_t$ | 94.0 | 90.0 |
| $F_b$—Pt—$F_t$ | 179.7 | 178.3 | Xe—$F_b$—Pt—$F_t$ | 46.5 | * |
| $F_b$—Pt—$F_t$ | 86.2 | 90.0 | Xe—$F_b$—Pt—$F_t$ | –46.6 | * |
| $F_b$—Pt—$F_b$ | 93.6 | 90.0 | Xe—$F_b$—Pt—$F_t$ | 47.0 | * |

† Crystal-structure data are reported as the average of all equivalent distances or angles in the symmetry-independent cations.

* A single averaged value is not reported because averaging the individual torsion angles would obscure their substantial variability. All experimentally determined angles are listed in Tables S2 and S3.

**Table S12.** Calculated (PBE0-D3/aug-cc-pVQZ(-PP)) geometrical parameters of the $[PdF_3(XeF_2)_3]^+$ cation.

| Bond | Calculated distance (Å) | Mayer bond order | Experimental distance† (Å) | Bond | Calculated distance (Å) | Mayer bond order | Experimental distance† (Å) |
|---|---|---|---|---|---|---|---|
| Xe—$F_t$ | 1.910 | 0.68 | 1.920 | Pd—$F_b$ | 1.984 | 0.54 | 1.981 |
| Xe—$F_b$ | 2.171 | 0.20 | 2.159 | Pd—$F_b$ | 1.985 | 0.53 | 1.981 |
| Xe—$F_t$ | 1.910 | 0.68 | 1.920 | Pd—$F_b$ | 1.983 | 0.54 | 1.981 |
| Xe—$F_b$ | 2.171 | 0.19 | 2.159 | Pd—$F_t$ | 1.845 | 0.89 | 1.853 |
| Xe—$F_t$ | 1.909 | 0.68 | 1.920 | Pd—$F_t$ | 1.845 | 0.88 | 1.853 |
| Xe—$F_b$ | 2.172 | 0.19 | 2.159 | Pd—$F_t$ | 1.845 | 0.88 | 1.853 |

| Angle (°) | Calculated | Experimental† | Angle (°) | Calculated | Experimental† |
|---|---|---|---|---|---|
| $F_t$—Xe—$F_b$ | 177.3 | 177.7 | $F_b$—Pd—$F_t$ | 87.2 | 90.0 |
| $F_t$—Xe—$F_b$ | 177.3 | 177.7 | $F_b$—Pd—$F_t$ | 87.3 | 90.0 |
| $F_t$—Xe—$F_b$ | 177.4 | 177.7 | $F_b$—Pd—$F_t$ | 179.5 | 178.7 |
| Pd—$F_b$—Xe | 119.9 | 126.7 | $F_b$—Pd—$F_t$ | 179.5 | 178.7 |
| Pd—$F_b$—Xe | 119.9 | 126.7 | $F_b$—Pd—$F_t$ | 87.5 | 90.0 |
| Pd—$F_b$—Xe | 120. 0 | 126.7 | $F_b$—Pd—$F_t$ | 87.4 | 90.0 |
| $F_b$—Pd—$F_b$ | 92.3 | 90.0 | $F_t$—Pd—$F_t$ | 92.8 | 90.0 |
| $F_b$—Pd—$F_b$ | 92.3 | 90.0 | $F_t$—Pd—$F_t$ | 93.0 | 90.0 |
| $F_b$—Pd—$F_t$ | 87.4 | 90.0 | $F_t$—Pd—$F_t$ | 93.0 | 90.0 |
| $F_b$—Pd—$F_t$ | 179.6 | 178.7 | Xe—$F_b$—Pd—$F_t$ | 46.0 | ‡ |
| $F_b$—Pd—$F_t$ | 87.3 | 90.0 | Xe—$F_b$—Pd—$F_t$ | 46.6 | ‡ |
| $F_b$—Pd—$F_b$ | 92.3 | 90.0 | Xe—$F_b$—Pd—$F_t$ | –45.3 | ‡ |

† Crystal-structure data are reported as the average of all equivalent distances or angles in the symmetry-independent cations.

‡A single averaged value is not reported because averaging the individual dihedral angles would mask the substantial variability they exhibit. All experimentally determined angles are listed in Table S4.

**Table S13.** Calculated AIM charges and Mayer valences for the $[MF_3(XeF_2)_3]^+$ cations and free $XeF_2$.

$[PtF_3(XeF_2)_3]^+$

| Atom | Pt | Xe | Xe | Xe | $F_t$(Xe) | $F_b$ | $F_t$(Xe) | $F_b$ | $F_t$(Xe) | $F_b$ | $F_t$(Pt) | $F_t$(Pt) | $F_t$(Pt) |
|---|---|---|---|---|---|---|---|---|---|---|---|---|---|
| AIM charge | 2.08 | 1.33 | 1.33 | 1.33 | −0.53 | −0.61 | −0.53 | −0.61 | −0.53 | −0.61 | −0.56 | −0.56 | −0.56 |
| Mayer valencies | 4.55 | 0.96 | 0.96 | 0.96 | 0.72 | 0.75 | 0.72 | 0.75 | 0.72 | 0.75 | 0.88 | 0.88 | 0.88 |

$[PdF_3(XeF_2)_3]^+$

| Atom | Pd | Xe | Xe | Xe | $F_t$(Xe) | $F_b$ | $F_t$(Xe) | $F_b$ | $F_t$(Xe) | $F_b$ | $F_t$(Pd) | $F_t$(Pd) | $F_t$(Pd) |
|---|---|---|---|---|---|---|---|---|---|---|---|---|---|
| AIM charge | 1.95 | 1.33 | 1.33 | 1.33 | −0.53 | −0.61 | −0.53 | −0.61 | −0.53 | −0.61 | −0.51 | −0.52 | −0.52 |
| Mayer valencies | 4.27 | 0.93 | 0.93 | 0.93 | 0.72 | 0.69 | 0.72 | 0.69 | 0.72 | 0.72 | 0.86 | 0.86 | 0.86 |

$XeF_2$

| Atom | Xe1 | F1 | F2 |
|---|---|---|---|
| AIM charge | 1.27 | −0.63 | −0.63 |
| Mayer valences | 1.12 | 0.64 | 0.64 |

Calculation of the molecular electrostatic potential surface (MEPS) after the removal of three $XeF_2$ units from the $[MF_3(XeF_2)_3]^+$ cations reveals pronounced σ-holes along the extensions of the M−F bonds (Figure S8). The maximum MEP value is slightly larger for $[PdF_3]^+$ (1027 kJ mol$^{-1}$) than for $[PtF_3]^+$ (1005 kJ mol$^{-1}$).

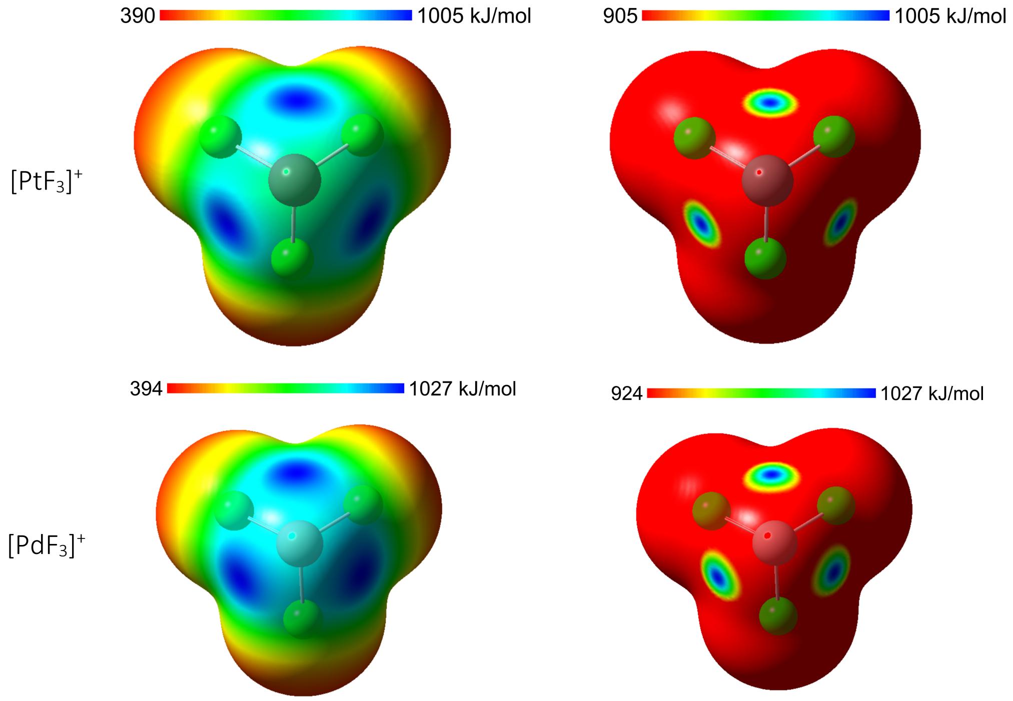


**Figure S8.** MEPS of $[MF_3]^+$ cations after removing the $XeF_2$ units (isoelectronic density value of 0.001 a.u.), shown using a color scale spanning the full potential range (left) or the top 10% of the positive range (right).

Relaxation of the $[MF_3]^+$ cation structures after removal of the $XeF_2$ ligands converges to virtually trigonal planar geometries. Calculation of the MEPS reveals regions of positive MEP at the extensions of the M−F bonds, which are positioned between the atoms. In this case, the maximum MEP is slightly higher for $[PtF_3]^+$ (730 kJ mol$^{-1}$) than for $[PdF_3]^+$ (723 kJ mol$^{-1}$).

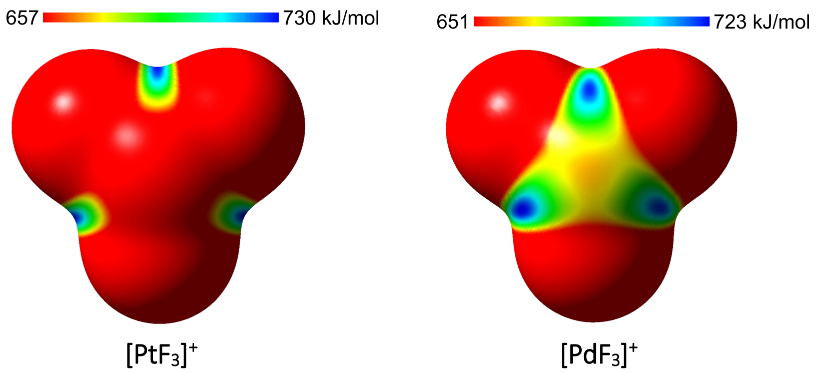


**Figure S9.** MEPS of the $[MF_3]^+$ cations with optimized geometries (isoelectronic density value of 0.001 a.u.), shown using a color scale spanning the top 10% of the positive electrostatic potential range.

**Table S14.** Optimized geometries of $[MF_3]^+$ cations (PBE0-D3/aug-cc-pVQZ).

| Atom | *x* | *y* | *z* |
|---|---|---|---|
| Pt | 7.258035 | 3.202891 | 8.978651 |
| F | 6.035872 | 3.733934 | 10.136923 |
| F | 7.885599 | 4.335574 | 7.778405 |
| F | 7.853036 | 1.541134 | 9.022856 |

| Atom | *x* | *y* | *z* |
|---|---|---|---|
| Pd | 3.129460 | 8.144106 | 15.164734 |
| F | 2.782243 | 8.825697 | 16.743463 |
| F | 4.343021 | 6.885260 | 15.036325 |
| F | 2.630131 | 9.019074 | 13.730110 |

**Table S15.** Optimized geometries of $[MF_6]^{2-}$ anions (PBE0-D3/aug-cc-pVQZ(-PP)).

| Atom | *x* | *y* | *z* |
|---|---|---|---|
| Pt | 2.254669 | 7.615310 | 0.000001 |
| F | 2.254670 | 9.563946 | 0.000000 |
| F | 4.203308 | 7.615314 | −0.000000 |
| F | 2.254667 | 7.615310 | 1.948641 |
| F | 0.306029 | 7.615317 | 0.000000 |
| F | 2.254668 | 7.615308 | −1.948640 |
| F | 2.254673 | 5.666665 | −0.000002 |

| Atom | *x* | *y* | *z* |
|---|---|---|---|
| Pd | 2.254670 | 7.615309 | 0.000001 |
| F | 2.254669 | 9.535551 | 0.000000 |
| F | 4.174911 | 7.615310 | 0.000000 |
| F | 2.254669 | 7.615311 | 1.920242 |
| F | 0.334428 | 7.615312 | −0.000000 |
| F | 2.254668 | 7.615309 | −1.920240 |
| F | 2.254670 | 5.695068 | −0.000002 |

**Table S16.** Calculated AIM charges, Mayer valences, and Mayer bond orders for the $[MF_6]^{2-}$ anions.

| | Atom | AIM | Mayer valence | Bond | Mayer bond order |
|---|---|---|---|---|---|
| $[PtF_6]^{2-}$ | Pt | 2.09 | 4.59 | Pt—F | 0.76 |
| | F | −0.68 | 0.64 | | |
| $[PdF_6]^{2-}$ | Pd | 1.97 | 4.19 | Pd—F | 0.70 |
| | F | −0.66 | 0.59 | | |

**Table S17.** QTAIM topological analysis of $[MF_3(XeF_2)_3]^+$ cations and free $XeF_2$: the electron density (ρ(*r*)), Laplacian of the electron density ($\nabla^2\rho(r)$), Lagrangian of kinetic energy (*G*(*r*)), potential energy density (*V*(*r*)), local energy density (*H*(*r*)), and electron localization function (ELF) were calculated at the bond critical point.

| Bond | Compound | $d_{SCXRD}$ (Å) | $d_{DFT}$ (Å) | Mayer order | ρ(*r*) (e Å$^{-3}$) | $\nabla^2\rho(r)$ (e Å$^{-5}$) | *G*(*r*) (kJ mol$^{-1}$) | *V*(*r*) (kJ mol$^{-1}$) | *H*(*r*) (kJ mol$^{-1}$) | ELF |
|---|---|---|---|---|---|---|---|---|---|---|
| M−$F_b$ | $[Pt(XeF_2)_3F_3]^+$ | 1.989 | 2.000 | 0.58 | 0.73 | 13.24 | 445.4 | −539.5 | −94.06 | 0.15 |
| | $[Pd(XeF_2)_3F_3]^+$ | 1.982 | 1.984 | 0.53 | 0.68 | 12.76 | 420.0 | −492.6 | −72.57 | 0.14 |
| M−$F_t$ | $[Pt(XeF_2)_3F_3]^+$ | 1.889 | 1.878 | 0.92 | 1.10 | 14.71 | 593.30 | −802.60 | −209.30 | 0.27 |
| | $[Pd(XeF_2)_3F_3]^+$ | 1.853 | 1.845 | 0.88 | 1.08 | 14.70 | 584.16 | −768.15 | −183.99 | 0.27 |
| Xe−$F_b$ | $[Pt(XeF_2)_3F_3]^+$ | 2.199 | 2.186 | 0.20 | 0.54 | 4.49 | 189.0 | −255.8 | −66.77 | 0.26 |
| | $[Pd(XeF_2)_3F_3]^+$ | 2.159 | 2.171 | 0.20 | 0.56 | 4.60 | 196.8 | −268.4 | −71.63 | 0.26 |
| Xe−$F_t$ | $[Pt(XeF_2)_3F_3]^+$ | 1.908 | 1.909 | 0.68 | 1.04 | 5.64 | 410.7 | −667.6 | −256.9 | 0.40 |
| | $[Pd(XeF_2)_3F_3]^+$ | 1.921 | 1.910 | 0.68 | 1.04 | 5.71 | 413.0 | −670.6 | −257.6 | 0.39 |
| Xe−F | $XeF_2$ | 1.999 | 1.982 | 0.56 | 0.88 | 5.36 | 335.2 | −524.4 | −189.2 | 0.36 |

**Table S18.** Observed bands in the low-temperature Raman spectra of $[Xe_2F_3][PtF_3(XeF_2)_3][AsF_6]_2$(*oP*256), $[Xe_2F_3][PtF_3(XeF_2)_3][AsF_6]_2$(*aP*256), and $[Xe_2F_3][PdF_3(XeF_2)_3][AsF_6]_2$ (Figure 3) with their relative intensities denoted in parentheses.

| $[Xe_2F_3][PtF_3(XeF_2)_3][AsF_6]_2$(*oP*256) Δν ($cm^{-1}$) | $[Xe_2F_3][PtF_3(XeF_2)_3][AsF_6]_2$(*aP*256) Δν ($cm^{-1}$) | $[Xe_2F_3][PdF_3(XeF_2)_3][AsF_6]_2$ Δν ($cm^{-1}$) |
|---|---|---|
| 62 (13) | 62 (19) | 60 (18) |
| 76 (11) | 73 (18) | 89 (13) |
| 86 (12) | 125 (1) | 120 (4) |
| 114 (2) | 157 (18) | 130 (7) |
| 129 (3) | 177 (sh) | 154 (26) |
| 153 (18) | 210 (7) | 163 (22) |
| 214 (5) | 216 (7) | 188 (2) |
| 224 (6) | 239 (2) | 227 (16) |
| 253 (1) | 245 (2) | 254 (11) |
| 295 (1) | 306 (4) | 270 (6) |
| 359 (10) | 352 (8) | 296 (3) |
| 369 (13) | 370 (11) | 345 (3) |
| 398 (1) | 407 (4) | 369 (19) |
| 407 (1) | 503 (1) | 394 (1) |
| 425 (1) | 518 (1) | 410 (6) |
| 490 (1) | 579 (39) | 478 (3) |
| 519 (1) | 590 (84) | 507 (4) |
| 576 (24) | 599 (100) | 587 (66) |
| 592 (80) | 635 (18) | 594 (100) |
| 599 (100) | 642 (26) | 599 (94) |
| 633 (8) | 648 (37) | 608 (35) |
| 642 (30) | 681 (20) | 636 (78) |
| 682 (23) | 691 (2) | 641 (sh) |
| 700 (13) | 704 (1) | 649 (sh) |
|  | 719 (1) | 681 (33) |

sh: shoulder

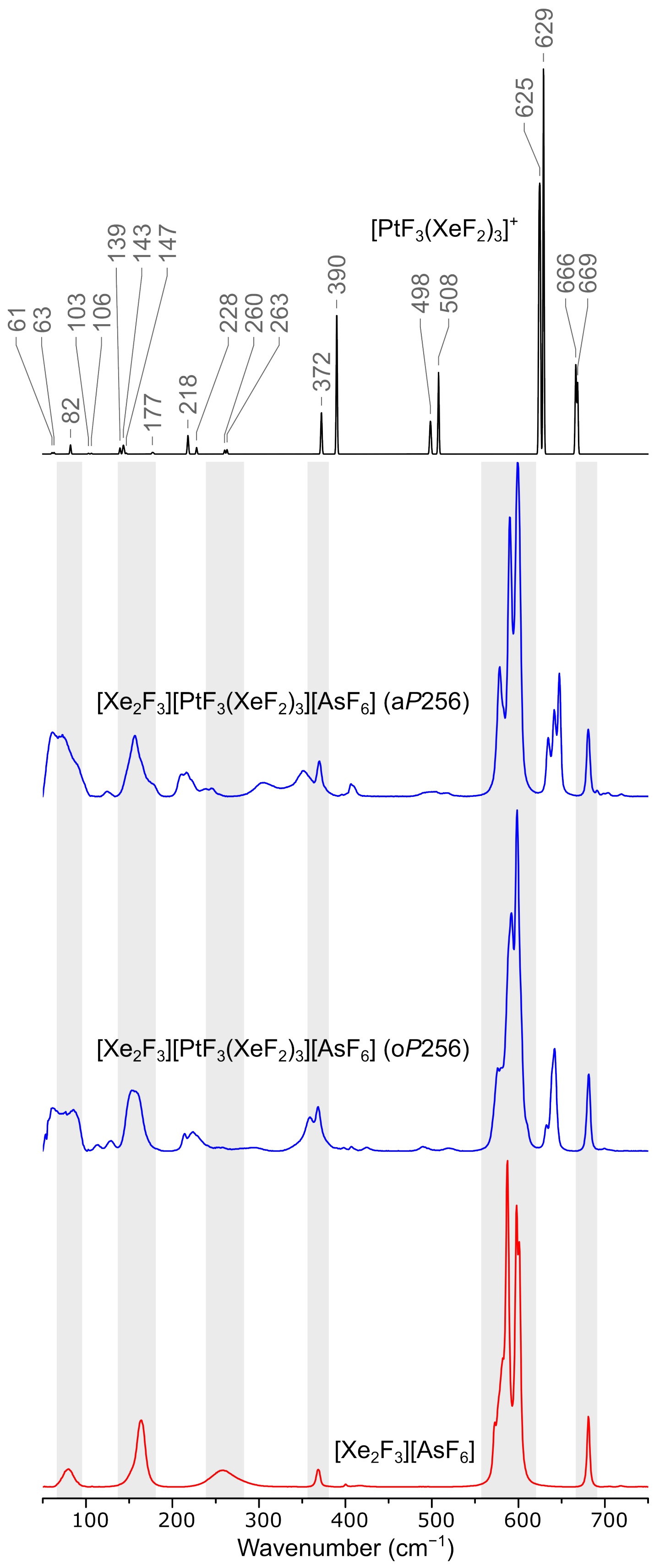


**Figure S10.** Comparison of the calculated Raman spectrum of $[PtF_3(XeF_2)_3]^+$ cation and experimentally measured Raman spectra of the two polymorphs of $[Xe_2F_3][PtF_3(XeF_2)_3][AsF_6]_2$ and $[Xe_2F_3][AsF_6]$. The shaded area highlights the bands observed in the Raman spectrum of $[Xe_2F_3][AsF_6]$.

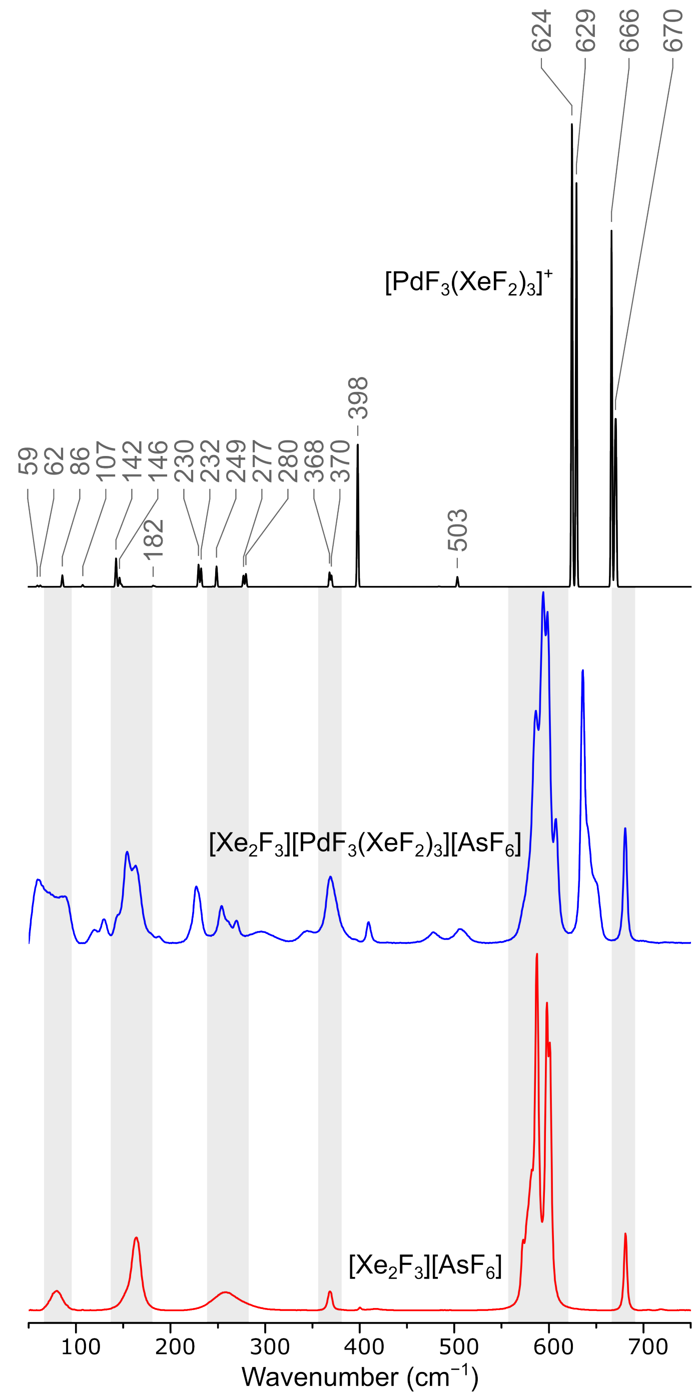


**Figure S11.** Comparison of the calculated Raman spectrum of $[PdF_3(XeF_2)_3]^+$ cation and experimentally measured Raman spectra of $[Xe_2F_3][PdF_3(XeF_2)_3][AsF_6]_2$ and $[Xe_2F_3][AsF_6]$. The shaded area highlights the bands observed in the Raman spectrum of $[Xe_2F_3][AsF_6]$.

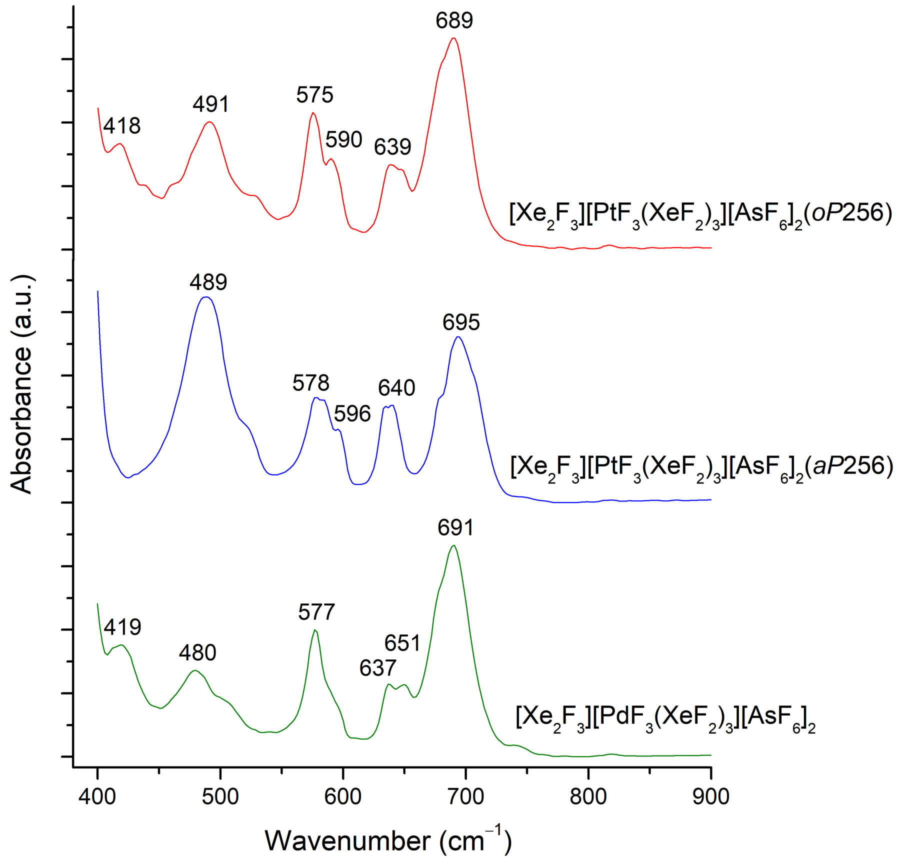


**Figure S12.** ATR-IR spectra recorded on powdered samples containing $[Xe_2F_3][PdF_3(XeF_2)_3][AsF_6]_2$(*oP*256), $[Xe_2F_3][PtF_3(XeF_2)_3][AsF_6]_2$(*aP*256), $[Xe_2F_3][PdF_3(XeF_2)_3][AsF_6]_2$, as well as $[Xe_2F_3][AsF_6]$ and $KAsF_6$ impurities.

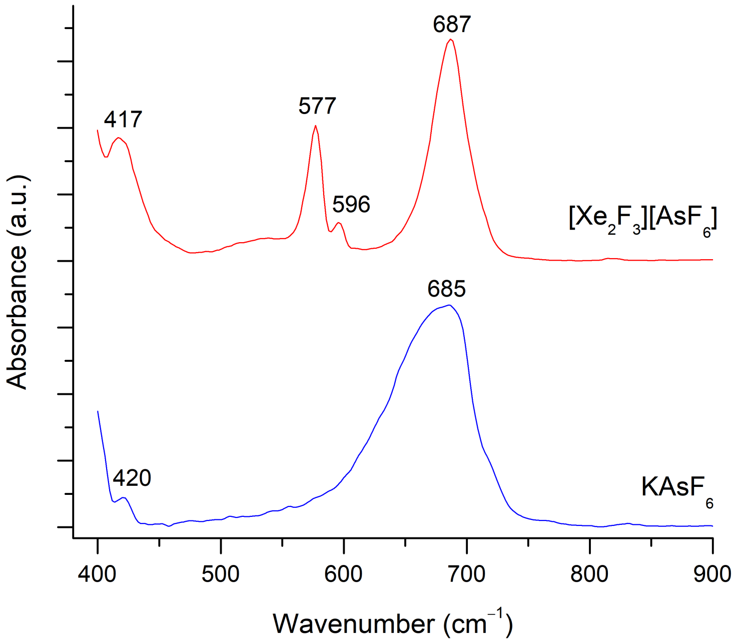


**Figure S13.** ATR-IR spectra of $[Xe_2F_3][AsF_6]$ and $KAsF_6$.

# References


(1) Alvarez, S. A Cartography of the van Der Waals Territories. *Dalton Trans.* **2013**, *42* (24), 8617–8636. https://doi.org/10.1039/C3DT50599E.

(2) Vogt, J.; Alvarez, S. Van Der Waals Radii of Noble Gases. *Inorg. Chem.* **2014**, *53* (17), 9260–9266. https://doi.org/10.1021/ic501364h.
